# Effects of Low and High Aneurysmal Wall Shear Stress on Endothelial Cell Behavior: Differences and Similarities

**DOI:** 10.3389/fphys.2021.727338

**Published:** 2021-10-14

**Authors:** Sandrine Morel, Sabine Schilling, Mannekomba R. Diagbouga, Matteo Delucchi, Marie-Luce Bochaton-Piallat, Sylvain Lemeille, Sven Hirsch, Brenda R. Kwak

**Affiliations:** ^1^Department of Pathology and Immunology, Faculty of Medicine, University of Geneva, Geneva, Switzerland; ^2^Neurosurgery Division, Department of Clinical Neurosciences, Faculty of Medicine, Geneva University Hospitals, Geneva, Switzerland; ^3^Institute of Applied Simulation, Zurich University of Applied Sciences, Wädenswil, Switzerland; ^4^Institute of Tourism and Mobility, Lucerne School of Business, Lucerne University of Applied Sciences and Arts, Lucerne, Switzerland

**Keywords:** intracranial aneurysm, endothelial cell, wall shear stress, cell shape, differential gene expression, cytoskeleton

## Abstract

**Background:** Intracranial aneurysms (IAs) result from abnormal enlargement of the arterial lumen. IAs are mostly quiescent and asymptomatic, but their rupture leads to severe brain damage or death. As the evolution of IAs is hard to predict and intricates medical decision, it is essential to improve our understanding of their pathophysiology. Wall shear stress (WSS) is proposed to influence IA growth and rupture. In this study, we investigated the effects of low and supra-high aneurysmal WSS on endothelial cells (ECs).

**Methods:** Porcine arterial ECs were exposed for 48 h to defined levels of shear stress (2, 30, or 80 dyne/cm^2^) using an Ibidi flow apparatus. Immunostaining for CD31 or γ-cytoplasmic actin was performed to outline cell borders or to determine cell architecture. Geometry measurements (cell orientation, area, circularity and aspect ratio) were performed on confocal microscopy images. mRNA was extracted for RNAseq analysis.

**Results:** ECs exposed to low or supra-high aneurysmal WSS were more circular and had a lower aspect ratio than cells exposed to physiological flow. Furthermore, they lost the alignment in the direction of flow observed under physiological conditions. The effects of low WSS on differential gene expression were stronger than those of supra-high WSS. Gene set enrichment analysis highlighted that extracellular matrix proteins, cytoskeletal proteins and more particularly the actin protein family were among the protein classes the most affected by shear stress. Interestingly, most genes showed an opposite regulation under both types of aneurysmal WSS. Immunostainings for γ-cytoplasmic actin suggested a different organization of this cytoskeletal protein between ECs exposed to physiological and both types of aneurysmal WSS.

**Conclusion:** Under both aneurysmal low and supra-high WSS the typical arterial EC morphology molds to a more spherical shape. Whereas low WSS down-regulates the expression of cytoskeletal-related proteins and up-regulates extracellular matrix proteins, supra-high WSS induces opposite changes in gene expression of these protein classes. The differential regulation in EC gene expression observed under various WSS translate into a different organization of the ECs’ architecture. This adaptation of ECs to different aneurysmal WSS conditions may affect vascular remodeling in IAs.

## Introduction

Intracranial aneurysms (IAs) resulting from the deformation and enlargement of the lumen in arteries of the circle of Willis affect three to five percent of the population (Lawton and Vates, 2017). Most of the IAs are quiescent and asymptomatic, but their rupture leads to severe brain damage or death. Decision to treat an unruptured IA has to be taken considering (1) the annual rupture rate of the IA which is depending of patient and aneurysm characteristics, and (2) the mortality and morbidity rates associated with the treatment of the IA (Greving et al., 2014; Etminan et al., 2015). Despite intense research it is today still impossible to precisely predict the rupture probability of an individual IA. To better forecast the evolution of an unruptured IA, a broad understanding of the IA pathophysiology is needed.

Biomechanical forces are considered as important actors in IA initiation, growth and rupture (Munarriz et al., 2016; Diagbouga et al., 2018). Wall shear stress (WSS) defined as the tangential force per unit area imposed by the blood flow on the arterial wall per unit area is sensed by endothelial cells (ECs). In cerebral arteries, optimal function of ECs is associated with a physiological WSS of 20–30 dyne/cm^2^ (Zhao et al., 2015). Under laminar shear stress, cells are aligned in the direction of the flow and are in a quiescent and cytoprotective state (Kwak et al., 2014). Whereas the role of high WSS gradients on the initiation of IA formation is well established, the impact of WSS on IA growth and rupture is less understood (Meng et al., 2014; Staarmann et al., 2019). Flow patterns change in evolving IAs depending on their location, geometry (especially IA neck dimension) and their parental artery’s geometry. In human IAs, low WSS close to 2 dyne/cm^2^ is typically observed in wide-neck aneurysms with a slow recirculating flow whereas supra-high WSS (> 70 dyne/cm^2^) is found in aneurysms with impinging jet flow (Meng et al., 2014; Munarriz et al., 2016; Staarmann et al., 2019). These aneurysmal flow patterns affect IA pathophysiology: while low WSS seems to promote atherosclerotic, inflammatory and thrombotic processes, supra-high WSS has been associated with smooth muscle cell phenotypic changes and apoptosis (Meng et al., 2014; Munarriz et al., 2016; Cebral et al., 2019; Frosen et al., 2019; Staarmann et al., 2019).

Both low and supra-high WSS can alter normal endothelium toward a dysfunctional state, a process called “endothelial injury.” In the past two decades, many studies revealed that ECs are able to detect and to respond in a specific manner to defined changes in shear stress magnitude, frequency or direction, this way dictating vascular pathophysiology (Kwak et al., 2014; Jiang et al., 2015). For instance, vulnerable or stable atherosclerotic lesions could be induced in mouse carotid arteries *in vivo* in response to low laminar or oscillatory WSS, respectively, using a shear stress-modifying cuff (Cheng et al., 2006). ECs are equipped with a multitude of potential shear stress sensors including membrane-associated molecules (e.g., ion channels, receptors, adhesion molecules), the glycocalyx and specific membrane microdomains such as caveolae and primary cilia (Ando and Yamamoto, 2013; Kwak et al., 2014). We recently reported that the EC response to aneurysmal low WSS was dampened in ECs without primary cilium, leading to disorganized intercellular junctions and increased endothelial permeability (Diagbouga et al., 2021). Altered endothelial function is supposed to be an important factor for the increased severity of IA disease observed in polycystic kidney disease (PKD) patients, a genetic disorder affecting primary cilia (Cho et al., 2021). Little is known, however, on the potential differences in endothelial dysfunction/injury induced by the diverse flow magnitudes in IAs. In the present study, we investigated the changes in EC gene expression and cellular morphology following exposure to low or supra-high WSS.

## Materials and Methods

### Cell Culture and Flow Experiments

Coronary arteries of 8-month-old pigs were obtained from a nearby slaughterhouse. Primary arterial ECs were isolated as previously described (Hao et al., 2002) and cultured in Dulbecco’s Modified Eagle Medium (DMEM) supplemented with 10% fetal calf serum and 100 g/mL heparin. Cells were grown in dishes coated with 1% gelatin (Sigma-Aldrich) at 37°C in a humidified atmosphere containing 5% CO_2_. For flow experiments, cells were seeded on 1% gelatin coated Ibidi μ-Slides VI^0.4^ at 30.000 cells per channel and grown until confluence. Laminar shear stress of 2, 30, or 80 dyne/cm^2^ was applied for 48 h using an Ibidi flow system as previously described (Pfenniger et al., 2012; Denis et al., 2019; Diagbouga et al., 2021).

### Immunofluorescence

Cultured cells were fixed in ice-cold 100% methanol for 5 min and stained using primary antibodies recognizing CD31 (endothelial cell membrane receptor, Santa Cruz sc-1506, 1/50) or γ-cytoplasmic actin (mAb 2A3, 1/100) (Dugina et al., 2009) for 2 h at room temperature. Next, Alexa Fluor 568-conjugated anti-goat and 488-conjugated anti-mouse antibodies (Thermo Fisher Scientific, 1/2000) were used for signal detection. Nuclei were counterstained with 4′,6-diamidino-2-phenylindol (DAPI). Fluorescence was preserved using Ibidi mounting medium. Images were obtained by confocal laser scanning microscopy (Leica SP5 or Zeiss LSM800 Airyscan). Images were analyzed using the NIH Image software (NIH AutoExtractor 1.51; National Institutes of Health).

### Quantifying Cell Shape Changes and Cell Orientation

The angle γ between the blood flow direction, which is for the remainder of the paper to be assumed to be in the positive *x*-axis direction, and each cell’s major axis characterized the cell’s orientation. Measured angles were between 0° and 180°. As the cell’s major axis orientation should be independent of an angle measurement in clockwise or anti-clockwise direction, we projected all angles γ‘ from the second quadrant to the first quadrant *via* γ = 180°−γ‘. We introduced the circularity measure C = 4πA/L^2^, with A for the area of the cell and L its perimeter. Whereas this measure is 1 for a perfectly circular cell, it is smaller than 1 for all other cell shapes. Cell elongation was quantified by the aspect ratio of the cell’s fitted ellipse. That is, the ratio of major axis to minor axis. All geometry measurements based on confocal microscopy of porcine arterial ECs were performed with the imaging processing package Fiji (Schindelin et al., 2012).

**FIGURE 1:**
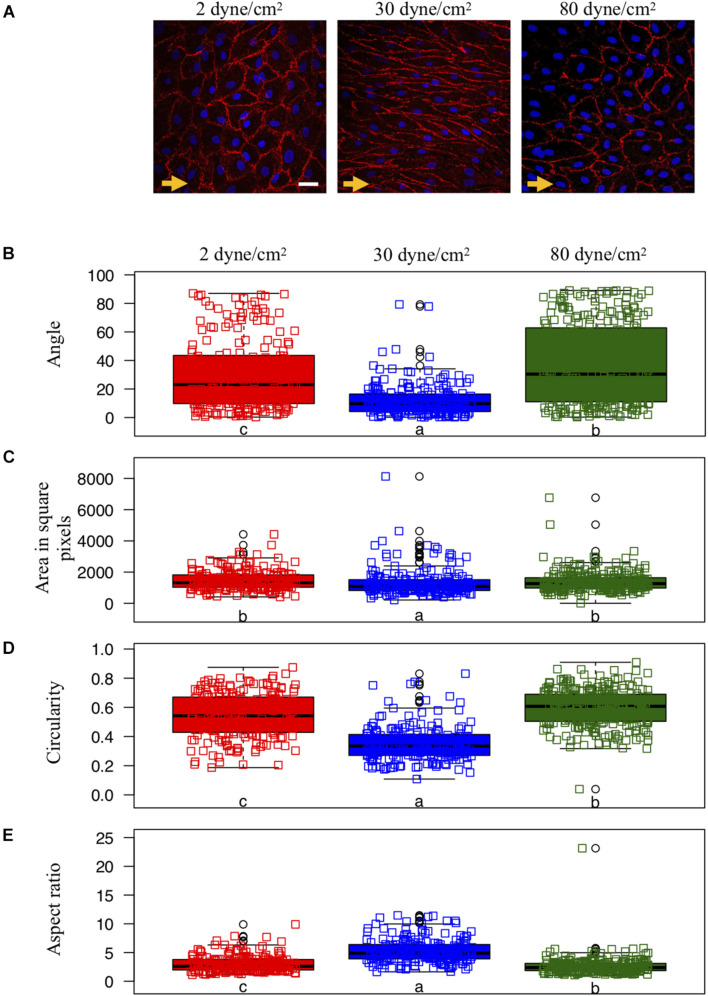
Endothelial cell shape and organization under various shear stress conditions. **(A)** Representative examples of ECs exposed to different *in vitro* conditions of shear stress: aneurysmal low shear stress = 2 dyne/cm^2^ (left panel), physiological shear stress = 30 dyne/cm^2^ (middle panel) or aneurysmal supra-high shear stress = 80 dyne/cm^2^ (right panel). The direction of the flow is indicated by orange arrows. CD31 immunostaining (in red) denotes the cell borders. Nuclei were stained with DAPI (in blue). Scale bar represents 20 μm. **(B–E)** Quantification of EC shapes parametrized by the angle between each cell’s major axis and flow **(B)**, the area **(C)**, the circularity **(D)** and the aspect ratio **(E)**. ECs were exposed to low (*N* = 208), physiological (*N* = 195) or supra-high (*N* = 271) conditions of shear stress. Results are shown as individual values and as boxed plots ± interquartile range. Two box plots sharing the same letter show no statistically significant difference (*p* < 0.01). Data points located above or below the box plot’s whiskers are considered outliers, defined as 1.5 times above or below the interquartile range, and marked by black circles.

We assumed the null hypothesis that the samples in the three different flow conditions for each cell shape measure originate from the same distribution. Assuming an error probability of α = 0.01 we rejected the null hypothesis for all three quantifications performing Kruskal-Wallis-tests resulting in significant *p*-values (*p* < 10^–6^). Subsequently, we performed for each shape measure pairwise two-sided Wilcoxon rank-sum-tests between the flow conditions with the Holm-Bonferoni correction for multiple testing (Holm, 1979). If the difference between two flow conditions was significant (*p* < 0.01), the small characters below the box plots in Figure 1 differ. All statistical tests were performed with the package visStatistics (Schilling, 2021) using the statistical software R (R Core Team, 2020).

### Library Preparation, Sequencing, Read Mapping to the Reference Genome

Total RNA was isolated from porcine arterial ECs using the nucleospin RNA II kit (Machery-Nagel) according to the manufacturers’ instructions. Three independent experiments were performed for each WSS condition, systematically comparing the WSS 2 dyne/cm^2^ vs. 30 dyne/cm^2^ or 80 dyne/cm^2^ vs. 30 dyne/cm^2^ from the same donor and cell passage. The quality of all samples was verified using the Agilent 2100 Bioanalyzer with the Agilent RNA 6000 Nano Kit (Agilent Technologies). cDNA libraries were constructed by the genomic platform of the University of Geneva using the Illumina TruSeq RNA sample preparation kit according to the manufacturers’ protocol. Libraries were sequenced using single-end (100 nt-long) on Illumina HiSeq2000. FastQ reads were mapped to the ENSEMBL reference genome (Sscrofa11.1.96) using STAR version 2.4.0j (Dobin et al., 2013) with standard settings, except that any reads mapping to more than one location of the genome (ambiguous reads) were discarded (*m* = 1). Sequence data have been submitted to GEO database under accession number GSE173928. A unique gene model was used to quantify reads per gene. Briefly, the model considers all annotated exons of all annotated protein coding isoforms of a gene to create a unique gene where the genomic region of all exons was considered as coming from the same RNA molecule and merged together.

### RNAseq Data Analysis

All reads overlapping the exons of each unique gene model were reported using featureCounts version 1.4.6-p1 (Liao et al., 2014). Gene expression was reported as raw counts and in parallel normalized in reads per kilobase million (RPKM) in order to filter out genes with low expression value (1 RPKM) before calling for differentially expressed genes. Library size normalizations and differential gene expression calculations were performed using the package edgeR (Robinson et al., 2010) designed for the R software (R Core Team, 2020). Only genes having a significant fold-change ≥ 2 and the Benjamini-Hochberg corrected *p*-value < 0.05 were considered for the differentially expressed genes analysis.

### Gene Set Enrichment Analysis

Families and sub-families of proteins were downloaded from PANTHER database for *Sus scrofa* and used to generate gene sets. Genes were ranked by their calculated fold-changes (decreasing ranking). A gene set analysis using the gene set enrichment analysis (GSEA) package Version 2.2 (Mootha et al., 2003; Subramanian et al., 2005) from the Broad Institute (MIT, Cambridge, MA) was used to analyze the pattern of differential gene expression between the two groups. Gene set permutations were performed 1,000 times for each analysis. The Normalized Enrichment Score (NES) was calculated for each gene set. GSEA results with a nominal False Discovery Rate (FDR) < 0.05 and abs(NES) > 1 were considered significant.

### PANTHER Gene Ontology

**FIGURE 2:**
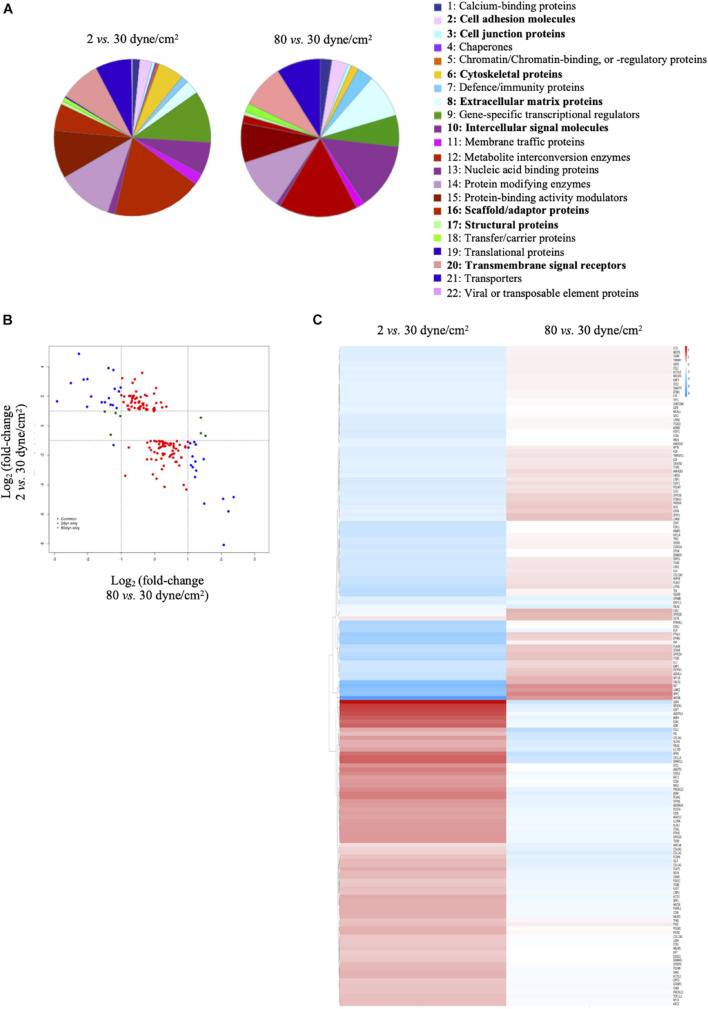
Low and supra-high shear stress affect protein classes distribution. **(A)** Pie charts of differentially expressed genes in ECs exposed to 2 dyne/cm^2^ vs. 30 dyne/cm^2^ (left panel) or 80 dyne/cm^2^ vs. 30 dyne/cm^2^ (right panel) based on Gene Ontology functional protein class. **(B)** Fold change expression level comparison of differentially expressed genes from the 8 protein classes written in bold in panel **(A)**. Red dots show genes modified in 2 vs. 30 dyne/cm^2^ only, green dots show genes modified in 80 vs. 30 dyne/cm^2^ only, and blue dots show genes modified in 2 vs. 30 dyne/cm^2^ and in 80 vs. 30 dyne/cm^2^. **(C)** Heat maps showing up- and down-regulation of common genes under 2 vs. 30 dyne/cm^2^ and 80 vs. 30 dyne/cm^2^.

Differentially expressed genes (either in 2 dyne/cm^2^ or 80 dyne/cm^2^ vs. 30 dyne/cm^2^) were annotated with the PANTHER tool gene ontology: protein class and represented in pie charts. 1: Calcium-binding proteins (PC00060); 2: Cell adhesion molecules (PC00069); 3: Cell junction proteins (PC00070); 4: Chaperones (PC00072); 5: Chromatin/chromatin-binding, or -regulatory proteins (PC00077); 6: Cytoskeletal proteins (PC00085); 7: Defense/immunity proteins (PC00090); 8: Extracellular matrix proteins (PC00102); 9: Gene-specific transcriptional regulators (PC00264); 10: Intercellular signal molecules (PC00207); 11: Membrane traffic proteins (PC00150); 12: Metabolite interconversion enzymes (PC00262); 13: Nucleic acid binding proteins (PC00171); 14: Protein modifying enzymes (PC00260); 15: Protein-binding activity modulators (PC00095); 16: Scaffold/adaptor proteins (PC00226); 17: Structural proteins (PC00211); 18: Transfer/carrier proteins (PC00219); 19: Translational proteins (PC00263); 20: Transmembrane signal receptors (PC00197); 21: Transporters (PC00227); 22: Viral or transposable element proteins (PC00237). Heatmaps were generated in R using the package pheatmap (Kolde, 2019). Figure 2C represents logFC (vs. respective control) for 2 dyne/cm^2^ and 80 dyne/cm^2^ conditions. Supplementary Figure 1 represents scaled RPKM values of genes of interest in replicates. For both heatmaps, genes were organized by hierarchical clustering using the function hclust in R.

## Results

### Endothelial Cell Shape and Orientation Are Altered Under Aneurysmal Wall Shear Stress Conditions

Porcine arterial ECs typically show a cobblestone morphology under static culture conditions [(Hao et al., 2002); not shown] and present an elongated shape and orientation in the direction of the flow after exposure for 48 h to a physiological WSS of 30 dyne/cm^2^ (Figure 1A—middle). However, ECs exposed to either type of aneurysmal WSS (2 or 80 dyne/cm^2^) failed to elongate (Figure 1A—left and right).

We quantified cell orientation and cell shape changes of the ECs under physiological WSS as well as under low and supra-high aneurysmal WSS by keeping track of each cell’s orientation, area, circularity and aspect ratio (Figures 1B–E). Whereas under physiological flow conditions, the ECs’ major axes were aligned toward the direction of flow (corresponding to an angle of 0°), this cell orientation was lost under both types of aneurysmal WSS (Figure 1B). Note that a completely random orientation of the cells would be reflected in an angle distribution centered around 45° and spread over the whole angle range between 0° and 90°. The cells’ surface area was smallest under physiological flow conditions and increased to a similar extend under both types of aneurysmal WSS (Figure 1C). Likewise, circularity was small under physiological flow conditions and increased under both types of aneurysmal WSS with highest value found at 80 dyne/cm^2^ (Figure 1D). Accordingly, the aspect ratio followed an inverted pattern with the highest value, denoting a high level of elongation, at the physiological WSS of 30 dyne/cm^2^ (Figure 1E). Thus, ECs lost their elongation and alignment in the direction of the flow, became roundish and increased their surface area under both types of aneurysmal flow conditions.

### Low and Supra-High Wall Shear Stress Differentially Affect Endothelial Cell Gene Expression

To obtain insight in the molecules involved in the endothelial shape changes observed in response to the different aneurysmal flow conditions, we performed unbiased transcriptomics (RNAseq) to compare gene expression levels in ECs exposed to physiological (30 dyne/cm^2^), low aneurysmal (2 dyne/cm^2^) or supra-high aneurysmal (80 dyne/cm^2^) WSS.

**FIGURE 3:**
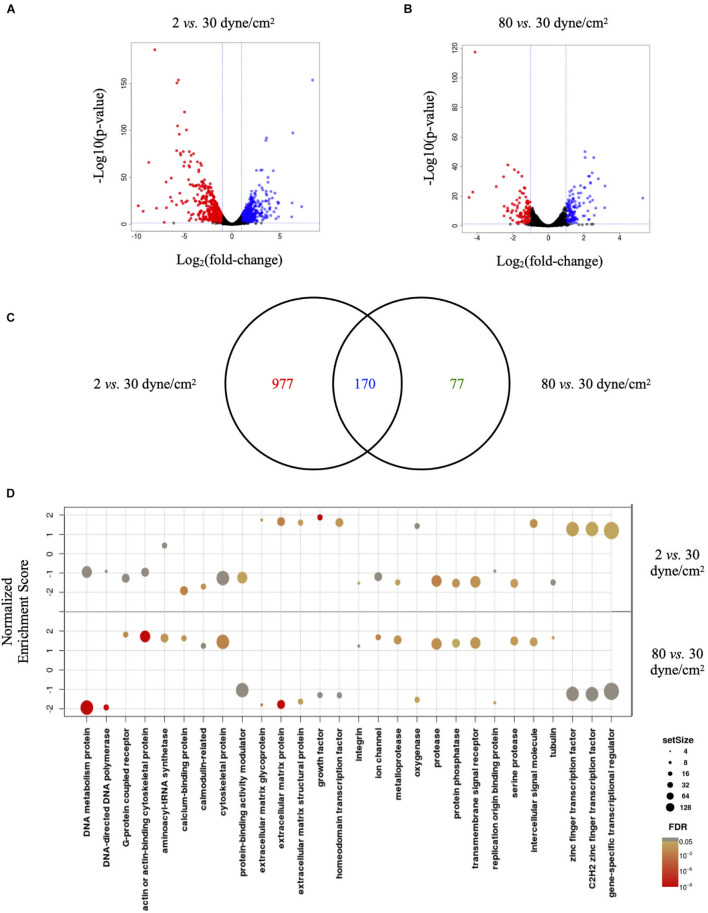
Low and supra-high shear stress affect endothelial cell genes expression. **(A,B)** Volcano plots displaying differential expressed genes in ECs under 2 vs. 30 dyne/cm^2^ **(A)** and 80 vs. 30 dyne/cm^2^ **(B)**. Blue and red dots represent the up- and down-regulated genes, respectively. **(C)** Venn diagram showing the number of shear stress-induced differentially expressed genes in ECs exposed to 2 vs. 30 dyne/cm^2^ or to 80 vs. 30 dyne/cm^2^. Colors represent differentially expressed genes in 2 dyne/cm^2^ (red), differentially expressed genes in 80 dyne/cm^2^ (green) or genes common between the two conditions (blue). **(D)** GSEA identifying essential pathways regulated by each aneurysmal WSS condition.

Visualizing the data in Volcano plots illustrates the divergence in magnitude of response when comparing low aneurysmal or supra-high aneurysmal WSS vs. physiological WSS. Indeed, when exposed to low aneurysmal shear stress, ECs showed approximately 5-fold more genes affected than when exposed to supra-high aneurysmal shear stress. The comparison of 2 dyne/cm^2^ to 30 dyne/cm^2^ identified 604 and 543 up- and down-regulated genes (Figure 3A and Supplementary Table 1). The same comparison between 80 dyne/cm^2^ and 30 dyne/cm^2^ yielded only 134 and 114 up- and down-regulated genes (Figure 3B and Supplementary Table 2). As illustrated in the Venn diagram of Figure 3C, 170 differentially expressed genes in low aneurysmal flow overlapped with differentially expressed genes in supra-high aneurysmal flow, indicating that they are regulated under pathological conditions independently of the magnitude of WSS. Moreover, 977 genes were uniquely affected under WSS of 2 dyne/cm^2^ and 77 genes were uniquely affected by WSS of 80 dyne/cm^2^. As an internal control, we compared the respective 30 dyne/cm^2^ physiological conditions with each other and revealed only 30 differentially expressed genes among the 10,446 tested genes (data not shown).

Next, we performed GSEA to identify essential pathways regulated by each aneurysmal WSS condition. Remarkably, GSEA performed in all up- and down-regulated genes showed that NES were inverted between 2 vs. 30 dyne/cm^2^ and 80 vs. 30 dyne/cm^2^ (Figure 3D). Whereas cytoskeletal proteins and more particularly actin or actin-binding proteins were among the classes the most up-regulated by supra-high WSS, these protein classes were down-regulated by low aneurysmal WSS. Conversely, various classes of extracellular matrix proteins were the most up-regulated by low WSS and these protein classes were down-regulated by pathological supra-high WSS.

To obtain further insight into biological functions of the differentially expressed genes, we performed a protein class functional annotation. Important differences were observed in protein classes known to determine cell shape and interactions, such as cell adhesion molecules, cell junction proteins, cytoskeletal proteins, extracellular matrix proteins, intercellular signal molecules, scaffold/adaptor proteins, structural proteins and transmembrane signal receptors (Figure 2A). A total of 208 genes in these 8 protein classes were differentially expressed between physiological and aneurysmal WSS (Supplementary Table 3), of which 159 and 14 were modified after the exposure to 2 dyne/cm^2^ or 80 dyne/cm^2^, respectively. The expression levels of 35 genes were affected by both types of aneurysmal WSS. Interestingly, the expression of these genes was regulated in an opposite direction by the pathological shear stresses (Figure 2B, blue dots). A heatmap representation comparing the alteration of gene expression in response to either aneurysmal WSS revealed an inverted regulation of nearly all differentially expressed genes (Figure 2C). The opposite gene expression patterns were observed in each of the three independent experiments with primary arterial ECs of different donors (Supplementary Figure 1), further emphasizing a consistent, robust and precise adaptation to different pathological conditions. Altogether, our results show that low and supra-high aneurysmal WSS induce important and opposite regulation of gene expression in ECs involving, among others, cytoskeletal and extracellular matrix proteins.

### Low and Supra-High Wall Shear Stress Differentially Affect Endothelial Cell γ-Cytoplasmic Actin Organization

**FIGURE 4:**
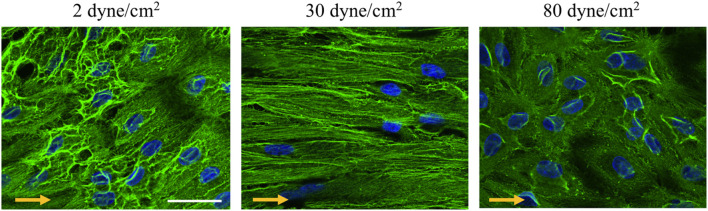
γ-cytoplasmic actin organization in endothelial cells exposed to different levels of shear stress. Representative examples of γ-cytoplasmic actin immunostainings (in green) performed on ECs exposed to 2 (left panel), 30 (middle panel) or 80 (right panel) dyne/cm^2^ for 48 h. Nuclei were stained with DAPI (in blue). Scale bar represents 20 μm. The direction of the flow is indicated by orange arrows.

Members of the actin protein family play critical roles in many aspects of eukaryotic cell biology, including cell shape maintenance, cell motility and cell contraction (Blanchoin et al., 2014). In vertebrates, six actin isoforms have been identified, each encoded by a different gene: two are typical of striated muscle (α-skeletal and α-cardiac actin), two are mainly found in smooth muscle (α- and γ-smooth muscle actin), and two are ubiquitous (β- and γ-cytoplasmic actin) (Dugina et al., 2009; Chaponnier and Gabbiani, 2016; Belvitch et al., 2018). To monitor the cells’ architecture in response to low and supra-high aneurysmal WSS, immunostainings were performed for γ-cytoplasmic actin in arterial ECs exposed to 2, 30 or 80 dyne/cm^2^ (Figure 4). In ECs exposed to the physiological flow of 30 dyne/cm^2^, γ-cytoplasmic actin formed an organized filamentous network, which was aligned in the direction of the flow (Figure 4—middle). Some features of the filamentous γ-cytoplasmic actin network were still observed in ECs exposed to low aneurysmal WSS, however, an increased density of γ-cytoplasmic actin filaments was observed in the peri-nuclear region of the ECs (Figure 4—left). In ECs exposed to supra-high WSS, γ-cytoplasmic actin filaments were preferentially found along the cell borders and in peri-nuclear regions (Figure 4—right). Together, these results indicate that the differential regulation in EC gene expression observed under various WSS translate into a different organization of the ECs’ architecture.

## Discussion

Initiation of IAs resulting in the deformation and enlargement of the arterial lumen is favored by high WSS and a high WSS gradient (WSSG) (Meng et al., 2014). Once IAs are formed, the aneurysm wall can be exposed to low, normal or supra-high values of WSS depending on the location and the geometry of the IA. Growing IAs change their geometry and as a result experience changing biomechanical forces over their life-time. The consequences of different and varying WSS for the evolution of IA disease are still poorly defined. In the present study, we show *in vitro* that ECs exposed to low or supra-high aneurysmal WSS were more circular than ECs exposed to physiological flow, and that they lost their alignment in the direction of the flow. We established that the effects of low WSS on differential gene expression were stronger than those of supra-high WSS. Moreover, low and supra-high aneurysmal WSS showed opposite regulation of gene expression in ECs involving cytoskeletal and extracellular matrix proteins. Finally, the differential regulation in EC gene expression observed under various pathological WSS translated into a different organization of the ECs’ architecture.

Hemodynamic forces play a key role in vascular physiology and pathophysiology. The mechanisms of how flow disturbances lead to endothelial dysfunction have been extensively studied in the context of atherosclerosis (Cheng et al., 2006; Kwak et al., 2014; Jiang et al., 2015). Little is known for other arterial diseases such as IAs, although a combination of computational fluid dynamics and *en face* immunofluorescence on human IAs has recently demonstrated increased activation of the pro-inflammatory transcription factor Nuclear Factor Kappa B in regions of oscillatory shear stress (OSS), similar to earlier studies involving atherosclerosis (Baeriswyl et al., 2019). Atherosclerotic lesions typically develop at arterial branch points or bifurcations, regions of the vascular tree exposed to OSS. The ECs in those regions are known to have a polygonal shape and a non-systematic positioning. Actin filaments in ECs of disturbed flow regions in general are shorter, irregular oriented and tend to cluster to the cells border (Kim et al., 1989). Cytoskeletal displacement or deformation in ECs has been proposed to be involved in the gene regulation toward a pro-inflammatory phenotype induced by OSS (Vartanian et al., 2010; Macek Jilkova et al., 2014; Speight et al., 2016). Similar to the architectural changes in arterial ECs exposed to OSS, we observed in our study a rearrangement of γ-cytoplasmic actin in ECs exposed to low or supra-high aneurysmal shear stress. The ECs rearranged from a highly organized filamentous network oriented in the direction of physiological blood flow to a disordered network clustered around their nuclei or alongside the cell edges with subtle but consistent differences between the two types of aneurysmal flow. Integrity of cell architecture is influenced by cytoskeletal proteins in which actin family play an important role (Pasquier et al., 2015; Dugina et al., 2019). Reorganization of cytoplasmic actins alters cell morphology. In fibroblasts and epithelial cells (Dugina et al., 2009), and more recently in endothelial cells (Pasquier et al., 2015), reduced expression of β- or γ-cytoplasmic actin have been shown to influence not only cell morphology, but also affect cell polarity, mobility or adhesion. In addition, the actin cytoskeleton has been proposed to provide a scaffold to stabilize the glycocalyx (Li and Wang, 2018). In our study, the differences in cytoskeletal rearrangement may stand at the basis for the differences in gene expression observed under low or supra-high aneurysmal shear stress.

Chronically elevated flow conditions, as observed in severely stenotic regions associated with atherosclerotic disease, are a well-known trigger for arterial remodeling (Gijsen et al., 2013; Munarriz et al., 2016). In an earlier study, DNA microarrays were used to profile EC gene expression in response to very high WSS (10 Pa; 100 dyne/cm^2^; 24 h) (Dolan et al., 2012). Compared to regular WSS (2 Pa; 20 dyne/cm^2^) or no-flow, very high WSS modulated gene expression in ways that promoted an anti-coagulant, anti-inflammatory, proliferative, and promatrix remodeling phenotypes, comprising genes essential for an adaptive expansive remodeling like *plasminogen activator, urokinase, tissue plasminogen activator, metalloproteinase ADAMTS1/6*, and *tissue inhibitor of metalloproteinase 3 (TIMP3)* (Dolan et al., 2012). In our study, *urokinase-type plasminogen activator*, *urokinase plasminogen activator surface receptor* and *ADAMTS* genes were down- and up-regulated under low and supra-high aneurysmal WSS, respectively. The expression of *TIMP3* was down-regulated under low aneurysmal WSS. These findings illustrate a tendency for more expansive remodeling in IAs exposed to supra-high aneurysmal WSS. In another study, Dolan et al. (2011) exposed ECs to IA initiating conditions using a flow chamber with constant-height channels to create regions of uniform (elevated) WSS and converging and diverging channels to create positive and negative WSSG, respectively. Similar to our study, highly elevated WSS (28.4 Pa; 284 dyne/cm^2^) inhibited EC alignment to flow (Dolan et al., 2011). In addition, increased EC proliferation and apoptosis was observed under elevated WSS, which was accentuated by a positive WSSG (+ 980 Pa/m) and suppressed by negative WSSG (–1120 Pa/m). The authors concluded that EC responses to positive WSSG may contribute to pathogenic remodeling that occurs at bifurcations preceding IA formation.

Although genetic predisposition has an important role in IA disease, little is known about the genetic architecture of IAs. Among the initial candidate genes discovered in gene expression and genome-wide linkage analyses are type III collagen (*COL3A1*), endoglin (*ENG*), fibrillin (*FBN1*), α1-antitrypsin (*SERPINA1*) and polycystin (*PKD1*, *PKD2*) (Nahed et al., 2007; Zhou et al., 2018), supporting observations of increased risk for IA formation in individuals with connective tissue disorders or PKD. A recent cross-ethnic genome-wide association study performed on 10,754 IA cases and 306,882 controls of European and East Asian ancestry has discovered a total of 17 risk loci of which 11 were new (Bakker et al., 2020). Many of these genes have known or putative roles in blood vessel physiology and blood pressure regulation, and an important role of ECs in IA development and rupture was proposed (Bakker et al., 2020). For some of these genes we found changes in the expression profiles. Locus 10q23.33 (rs11187838) and 15q25.1 (rs8034191) described in Bakker et al. (2020) and coding for the genes *PLCE1* (phospholipase C epsilon 1) and *HYKK* (hydroxylysine kinase) were up-regulated in ECs exposed to low vs. physiological WSS, whereas *STARD13* (StAR-related lipid transfer domain protein 13, locus 13q13.1, rs3742321) was down-regulated. STARD13 is a Rho GTPase-activating protein involved in regulation of cytoskeletal reorganization, cell proliferation, and cell motility. This is in line with our findings and supports the importance of these processes in the remodeling of IAs.

We have chosen to use primary porcine arterial ECs for this study, as this species is known to be very similar to humans with respect to cardiovascular anatomy, physiology, metabolism and disease (Crisostomo et al., 2016; Tsang et al., 2016). Moreover, porcine coronary EC isolation does not require the use of laboratory animals (3Rs principles). Even though in human the diameter of arteries in the circle of Willis resembles the one of coronary arteries (Dodge et al., 1992; Rai et al., 2013), the use of primary ECs isolated from porcine coronary arteries remains a limitation of our study as their properties may not completely recapitulate the ones of ECs in cerebral arteries. Moreover, both types of ECs are *in situ* exposed to different flow patterns and pressures. In this respect, it should also be noted that in our *in vitro* flow system ECs are exposed to a defined constant flow rather than to pulsatile flow as they experience *in vivo*. Finally, the porcine arterial ECs were cultured on 1% gelatin, a commonly used substrate for ECs in culture. A change in substrate may affect the basic properties of ECs and possibly induce subtle changes in their response to shear stress (Dessalles et al., 2021). Although we expect that the strong and opposite regulation in gene expression of cytoskeletal and extracellular matrix proteins observed in ECs exposed to low and supra-high aneurysmal WSS would be largely maintained on different substrates, this remains to be proven.

In addition of being exposed to shear stress caused by blood flow, ECs are subjected to cyclic strain caused by intravascular pressure, the level of which varies along the vascular tree. Under physiological conditions, the (elastic) human aorta undergoes about 10% increase in diameter, whereas (muscular) peripheral arteries undergo only 5% cyclic circumferential stretch (CCS) (Anwar et al., 2012). Physiological CCS is known to participate in vascular maintenance by regulating processes such as cell proliferation, morphology and extracellular matrix formation (Wang et al., 2003; Yoshigi et al., 2003; Li and Sumpio, 2005; Nishimura et al., 2006). The precise level of cyclic stretch in saccular IAs is unknown, but it is in general considered to be very low due to a combination of altered pulse waves and the low compliance of the IA wall. As the IA enlarges and its vessel wall becomes stiffer, ECs will thus gradually loose the cyclic stimulation required for their normal functioning. To this date, most studies on the response of ECs to CCS have focused on ECs exposed to high level of cyclic stretch as observed in hypertensive patients (Munarriz et al., 2016; Ando and Yamamoto, 2021) or at the prospective site of IA formation (Koseki et al., 2020) and very little is known onto the effects of reduced CCS on EC physiology. Such studies, and mostly studies in which various hemodynamic stresses are combined, remain one of the main challenges in the field of EC mechanobiology (Dessalles et al., 2021) but are expected to contribute greatly to our understanding of the pathogenesis of IAs and the prediction of IA rupture.

## Data Availability Statement

The code and dataset analyzed for the cell shape and orientation analysis are available on Github.com/hirshlab/aneurysmalWSSon EC, version 1.0 (https://github.com/hirsch-lab/aneurysmalWSSo nEC). RNA sequencing data have been submitted to the GEO database under accession number GSE173928 (https://www.ncbi.nlm.nih.gov/geo/query/acc.cgi?acc=GSE173928).

## Author Contributions

SM: acquisition of data, analysis of data, interpretation of analysis, and drafting of manuscript. SS: acquisition of data, analysis of data, interpretation of analysis, and revision of manuscript. MRD: acquisition of data and revision of manuscript. MD and SL: analysis of data and revision of manuscript. M-LB-P: materials provider and revision of manuscript. SH and BK: design and conceptualization of the study, interpretation of analysis, drafting of manuscript. All authors contributed to the article and approved the submitted version.

## Conflict of Interest

The authors declare that the research was conducted in the absence of any commercial or financial relationships that could be construed as a potential conflict of interest.

## Publisher’s Note

All claims expressed in this article are solely those of the authors and do not necessarily represent those of their affiliated organizations, or those of the publisher, the editors and the reviewers. Any product that may be evaluated in this article, or claim that may be made by its manufacturer, is not guaranteed or endorsed by the publisher.

## Supplementary Material

Supplementary Figure 1 Heatmap showing reproducibility of expression (in RPKM) of the differentially expressed genes. Results are shown for primary arterial ECs of 3 different donors submitted to 2 dyne/cm^2^ and 80 dyne/cm^2^.

Supplementary Table 1 Differentially expressed genes in ECs exposed to 2 dyne/cm^2^ vs. ECs exposed to 30 dyne/cm^2^.

Supplementary Table 2 Differentially expressed genes in ECs exposed to 80 dyne/cm^2^ vs. ECs exposed to 30 dyne/cm^2^.

Supplementary Table 3 Genes with an up- or down-regulated expression in the 8 following protein classes: Cell adhesion molecules, Cell junction proteins, Cytoskeletal proteins, Extracellular matrix proteins, Intercellular signal molecules, Scaffold/adaptor proteins, Structural proteins and Transmembrane signal receptors.
