# Supplementary figures and images for: Effects of Low and High Aneurysmal Wall Shear Stress on Endothelial Cell Behavior: Differences and Similarities
Source: Front Physiol. 2021 Oct 14;12:727338. doi: 10.3389/fphys.2021.727338 (PMC8551710; doi:10.3389/fphys.2021.727338)

2 dyne/cm<sup>2</sup>

80 dyne/cm<sup>2</sup>

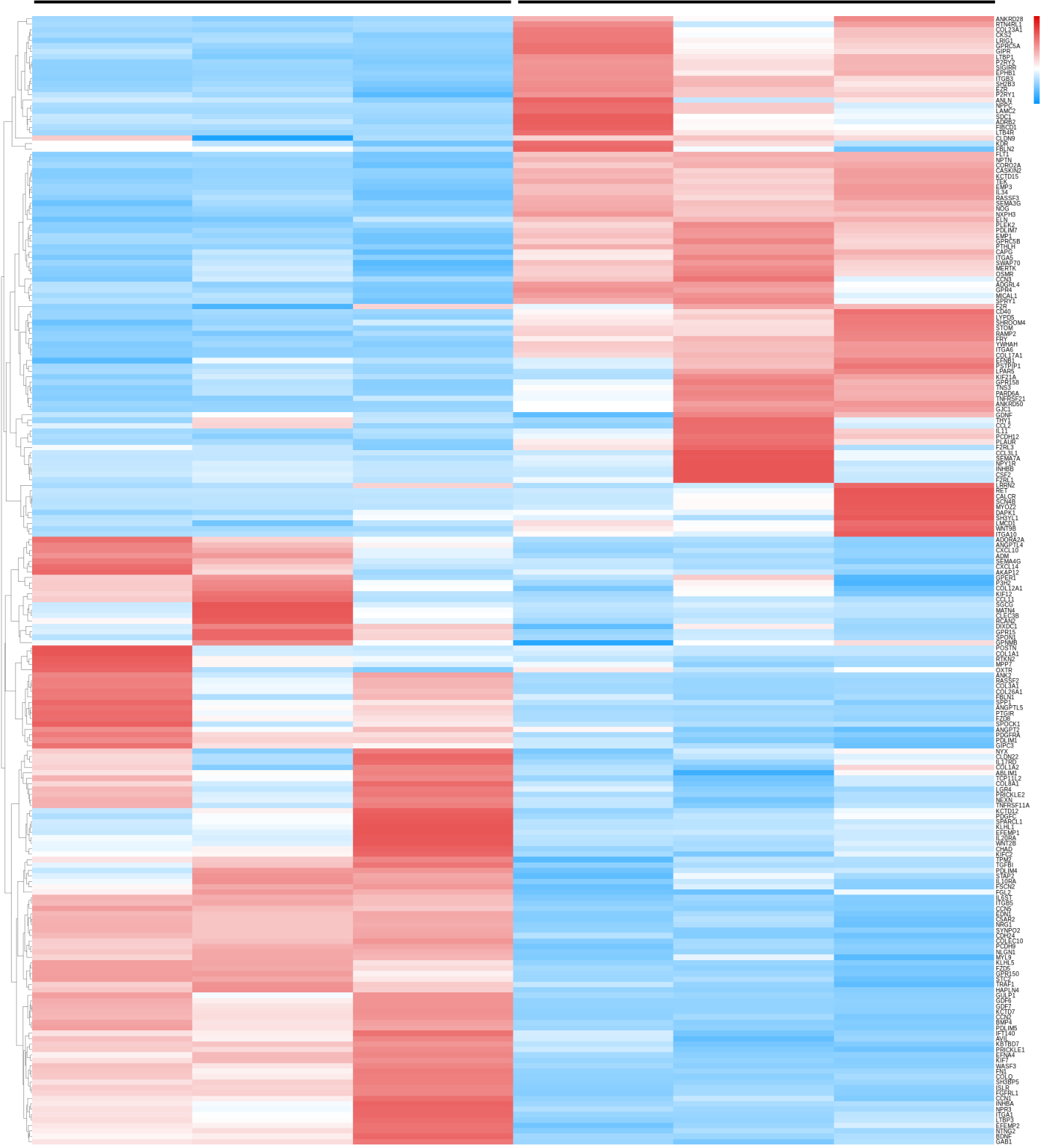

Supplement: Supplementary Figure 1 — Heatmap showing reproducibility of expression (in RPKM) of the differentially expressed genes. Results are shown for primary arterial ECs of 3 different donors submitted to 2 dyne/cm2 and 80 dyne/cm2. [file Data_Sheet_1.PDF]
