# Supplementary material for: Effects of Low and High Aneurysmal Wall Shear Stress on Endothelial Cell Behavior: Differences and Similarities
Source: Front Physiol. 2021 Oct 14;12:727338. doi: 10.3389/fphys.2021.727338 (PMC8551710; doi:10.3389/fphys.2021.727338)
Supplement: Supplementary Table 1 — Differentially expressed genes in ECs exposed to 2 dyne/cm2 vs. ECs exposed to 30 dyne/cm2. [file Data_Sheet_2.PDF]

Supplemental table 1: Differentially expressed genes in ECs exposed to 2 dyne/cm^2 *vs.* ECs exposed to 30 dyne/cm^2

**Gene_id**	**Gene_name**	**Log$_2$ fold change**	**P-value**
ENSSSCG00000003431	NPPB	-9,79	2,744E-20
ENSSSCG00000038956	HEYL	-9,29	2,337E-14
ENSSSCG00000010325	KCNMA1	-8,70	1,260E-66
ENSSSCG00000035524	WNT9B	-8,07	1,406E-186
ENSSSCG00000015320	CALCR	-7,89	1,164E-17
ENSSSCG00000037555	NA	-7,08	1,065E-02
ENSSSCG00000026067	TMPRSS6	-6,89	1,060E-45
ENSSSCG00000006183	SBSPON	-6,85	3,475E-18
ENSSSCG00000016295	NGEF	-6,42	2,260E-29
ENSSSCG00000010370	NA	-6,34	7,257E-50
ENSSSCG00000013976	NA	-6,28	4,132E-25
ENSSSCG00000010199	RET	-5,80	3,011E-21
ENSSSCG00000036801	C6orf132	-5,78	3,507E-79
ENSSSCG00000012026	ADAMTS1	-5,74	2,265E-151
ENSSSCG00000011557	CIDEC	-5,69	1,964E-105
ENSSSCG00000024158	ANO1	-5,59	2,057E-154
ENSSSCG00000016002	NA	-5,51	1,541E-96
ENSSSCG00000013403	GALNT18	-5,44	1,099E-33
ENSSSCG00000004889	SERPINB10	-5,39	2,323E-76
ENSSSCG00000003694	EMILIN2	-5,35	1,624E-74
ENSSSCG00000005707	FIBCD1	-5,27	2,744E-25
ENSSSCG00000028076	ZBTB7C	-5,12	8,832E-23
ENSSSCG00000006287	SELL	-5,09	3,147E-78
ENSSSCG00000010449	CH25H	-5,04	1,231E-62
ENSSSCG00000015556	LAMC2	-4,95	2,948E-120
ENSSSCG00000032374	SULT1B1	-4,94	5,559E-67
ENSSSCG00000026852	NPPC	-4,83	2,823E-24
ENSSSCG00000030388	UPP1	-4,76	3,979E-101
ENSSSCG00000006578	S100A4	-4,67	1,909E-25
ENSSSCG00000009000	NA	-4,53	3,201E-13
ENSSSCG00000009616	HR	-4,53	3,782E-48
ENSSSCG00000022256	DEPP1	-4,49	3,997E-63
ENSSSCG00000015323	GNGT1	-4,47	1,378E-04
ENSSSCG00000015383	RAPGEF5	-4,44	4,169E-78
ENSSSCG00000000261	IGFBP6	-4,42	1,060E-61
ENSSSCG00000038911	KLF2	-4,38	5,969E-43
ENSSSCG00000036893	PTHLH	-4,32	1,673E-67
ENSSSCG00000004644	HDC	-4,29	7,532E-07
ENSSSCG00000021259	CDA	-4,26	3,503E-74

ENSSSCG00000006595	IVL	-4,24	1,445E-10
ENSSSCG00000009338	FRY	-4,14	1,021E-42
ENSSSCG00000001989	CIDEB	-4,05	1,586E-11
ENSSSCG00000006472	CRABP2	-4,02	1,951E-41
ENSSSCG00000024492	EPHB1	-4,01	6,623E-76
ENSSSCG00000024837	SYT12	-3,97	2,957E-28
ENSSSCG00000015894	DPP4	-3,84	1,527E-25
ENSSSCG00000031321	NR4A1	-3,79	9,834E-42
ENSSSCG00000021515	HS3ST1	-3,78	2,956E-25
ENSSSCG00000023591	ADGRF2	-3,75	2,777E-41
ENSSSCG00000001490	KHDRBS2	-3,75	1,770E-05
ENSSSCG00000003600	TINAGL1	-3,71	4,737E-42
ENSSSCG00000024791	NA	-3,61	2,868E-67
ENSSSCG00000039780	RTN4RL1	-3,58	3,043E-32
ENSSSCG00000001570	PI16	-3,52	7,837E-21
ENSSSCG00000040110	NA	-3,52	3,959E-11
ENSSSCG00000017306	ITGB3	-3,47	1,206E-42
ENSSSCG00000016233	SERPINE2	-3,46	9,381E-57
ENSSSCG00000001926	NA	-3,46	5,310E-10
ENSSSCG00000002135	PNP	-3,45	9,249E-59
ENSSSCG00000006288	SELP	-3,41	7,111E-25
ENSSSCG00000023522	TGM2	-3,40	8,995E-39
ENSSSCG00000025858	ELN	-3,38	7,445E-07
ENSSSCG00000010497	ENTPD1	-3,38	2,419E-13
ENSSSCG00000003069	KCNN4	-3,33	1,307E-22
ENSSSCG00000004980	THSD4	-3,31	1,117E-12
ENSSSCG00000014091	F2RL1	-3,29	1,403E-23
ENSSSCG00000039758	NA	-3,29	4,303E-14
ENSSSCG00000025270	CHRD	-3,29	8,323E-66
ENSSSCG00000006579	S100A3	-3,28	3,938E-06
ENSSSCG00000022961	CLMP	-3,27	1,222E-14
ENSSSCG00000006533	ADAM15	-3,26	3,697E-41
ENSSSCG00000020837	NA	-3,20	7,269E-15
ENSSSCG00000010312	PLAU	-3,19	1,825E-63
ENSSSCG00000006359	ADAMTS4	-3,18	1,296E-36
ENSSSCG00000008491	QPCT	-3,17	4,741E-46
ENSSSCG00000013880	NA	-3,17	2,373E-27
ENSSSCG00000035224	NA	-3,10	4,525E-29
ENSSSCG00000033182	NA	-3,10	1,434E-03
ENSSSCG00000003582	SMPDL3B	-3,08	1,055E-28
ENSSSCG00000034440	MMP28	-3,07	3,195E-45
ENSSSCG00000015273	ATP2B4	-3,07	2,203E-42

ENSSSCG00000011596	TRH	-3,06	4,026E-46
ENSSSCG00000000188	DHH	-3,05	3,231E-30
ENSSSCG00000009182	NA	-3,04	7,767E-06
ENSSSCG00000029592	GPRC5A	-3,03	1,486E-40
ENSSSCG00000011600	SLC6A6	-3,03	1,581E-23
ENSSSCG00000011837	MELTF	-3,01	4,859E-09
ENSSSCG00000033286	NA	-3,01	2,399E-23
ENSSSCG00000035736	NA	-2,97	9,359E-08
ENSSSCG00000023909	THPO	-2,97	2,352E-11
ENSSSCG00000003839	NA	-2,96	1,318E-30
ENSSSCG00000005122	TEK	-2,95	4,143E-44
ENSSSCG00000003578	FGR	-2,92	3,382E-13
ENSSSCG00000029752	C16orf54	-2,92	9,190E-06
ENSSSCG00000002818	PLLP	-2,90	6,446E-23
ENSSSCG00000027677	NA	-2,88	5,434E-24
ENSSSCG00000024166	SLC2A6	-2,87	5,416E-13
ENSSSCG00000036136	BHLHE40	-2,87	5,052E-48
ENSSSCG00000033190	NA	-2,84	2,367E-38
ENSSSCG00000038618	RFLNB	-2,83	9,037E-06
ENSSSCG00000036575	USHBP1	-2,81	1,231E-39
ENSSSCG00000032434	PLAUR	-2,81	4,420E-28
ENSSSCG00000012006	NA	-2,81	4,933E-41
ENSSSCG00000030960	NA	-2,79	4,763E-08
ENSSSCG00000007572	LFNG	-2,76	3,148E-24
ENSSSCG00000034297	HES2	-2,75	7,960E-42
ENSSSCG00000024389	SIGIRR	-2,71	1,068E-24
ENSSSCG00000038196	NA	-2,70	3,290E-06
ENSSSCG00000021711	C16orf86	-2,70	9,237E-09
ENSSSCG00000003073	LYPD5	-2,69	9,290E-23
ENSSSCG00000017178	NA	-2,69	2,298E-28
ENSSSCG00000029199	SCN4B	-2,68	1,302E-06
ENSSSCG00000001518	ITPR3	-2,67	2,875E-30
ENSSSCG00000004053	TAGAP	-2,66	4,353E-20
ENSSSCG00000016548	NA	-2,66	1,044E-26
ENSSSCG00000025021	MALL	-2,63	2,498E-33
ENSSSCG00000006199	PREX2	-2,63	3,624E-30
ENSSSCG00000005437	NA	-2,62	4,547E-30
ENSSSCG00000015850	DUSP4	-2,62	1,093E-14
ENSSSCG00000006018	TRPS1	-2,53	4,548E-16
ENSSSCG00000004670	C15orf48	-2,52	1,140E-16
ENSSSCG00000011405	HYAL2	-2,51	1,122E-38
ENSSSCG00000040410	NA	-2,49	2,532E-35

ENSSSCG00000016031	CALCRL	-2,49	1,461E-40
ENSSSCG00000033844	CYP1B1	-2,47	6,093E-20
ENSSSCG00000031539	NA	-2,47	6,227E-05
ENSSSCG00000017552	NXPH3	-2,44	2,979E-11
ENSSSCG00000002290	PLEK2	-2,44	4,831E-40
ENSSSCG00000037418	NA	-2,44	1,076E-04
ENSSSCG00000032123	SPX	-2,44	2,673E-13
ENSSSCG00000003762	ADGRL4	-2,42	8,379E-35
ENSSSCG00000008038	SLC9A3R2	-2,41	9,448E-25
ENSSSCG00000031893	NA	-2,41	2,205E-36
ENSSSCG00000006580	S100A2	-2,40	1,040E-14
ENSSSCG00000028661	ENKD1	-2,38	6,217E-07
ENSSSCG00000006542	KCNN3	-2,35	3,414E-11
ENSSSCG00000022506	NA	-2,35	1,191E-09
ENSSSCG00000000837	CHST11	-2,34	1,312E-13
ENSSSCG00000004497	PSTPIP2	-2,34	9,237E-09
ENSSSCG00000015281	PLEKHA6	-2,33	3,024E-09
ENSSSCG00000017389	RAMP2	-2,31	2,142E-40
ENSSSCG00000016068	HECW2	-2,31	1,633E-15
ENSSSCG00000038616	NA	-2,30	1,302E-10
ENSSSCG00000017927	BCL6B	-2,30	5,889E-32
ENSSSCG00000007239	CCM2L	-2,28	6,679E-35
ENSSSCG00000013252	F2	-2,28	4,468E-18
ENSSSCG00000035078	CD40	-2,27	8,005E-44
ENSSSCG00000015930	DHRS9	-2,27	1,445E-06
ENSSSCG00000013868	F2RL3	-2,26	9,929E-10
ENSSSCG00000008888	NPY1R	-2,26	7,759E-12
ENSSSCG00000030241	TSC22D3	-2,25	8,143E-33
ENSSSCG00000007147	HSPA12B	-2,24	7,176E-25
ENSSSCG00000000779	KIF21A	-2,23	1,306E-09
ENSSSCG00000037935	CYYR1	-2,22	6,078E-26
ENSSSCG00000036824	AVPI1	-2,21	7,073E-19
ENSSSCG00000004666	NA	-2,19	1,866E-24
ENSSSCG00000015283	PIK3C2B	-2,18	1,625E-39
ENSSSCG00000027443	MRAS	-2,18	9,648E-32
ENSSSCG00000011904	UPK1B	-2,18	7,324E-10
ENSSSCG00000039419	SLCO4A1	-2,17	3,286E-18
ENSSSCG00000014029	COL23A1	-2,16	1,866E-24
ENSSSCG00000007866	TMC7	-2,16	6,915E-22
ENSSSCG00000036723	EMP1	-2,15	7,521E-18
ENSSSCG00000009233	GPAT3	-2,15	3,063E-11
ENSSSCG00000017380	ARL4D	-2,15	6,039E-05

ENSSSCG00000040725	IL11	-2,14	2,211E-07
ENSSSCG00000031154	ACKR2	-2,13	1,137E-10
ENSSSCG00000010606	SLK	-2,13	1,269E-24
ENSSSCG00000011511	FRMD4B	-2,11	1,233E-33
ENSSSCG00000040629	IL34	-2,10	2,035E-06
ENSSSCG00000011825	ATP13A3	-2,10	1,221E-31
ENSSSCG00000015664	NA	-2,10	1,435E-22
ENSSSCG00000001867	PSTPIP1	-2,08	3,547E-06
ENSSSCG00000034569	NA	-2,07	1,581E-10
ENSSSCG00000012546	NRK	-2,07	1,185E-07
ENSSSCG00000008318	VAX2	-2,07	6,071E-06
ENSSSCG00000006286	NA	-2,06	2,133E-05
ENSSSCG00000022925	NA	-2,06	6,396E-36
ENSSSCG00000001988	ADCY4	-2,06	2,272E-32
ENSSSCG00000016976	ZNF366	-2,06	1,508E-07
ENSSSCG00000033018	TM4SF1	-2,05	6,953E-17
ENSSSCG00000015550	RGS16	-2,04	1,047E-06
ENSSSCG00000003253	NA	-2,04	4,131E-25
ENSSSCG00000023374	SRGN	-2,04	2,167E-07
ENSSSCG00000015871	NR4A2	-2,04	1,534E-04
ENSSSCG00000011914	ZDHHC23	-2,03	7,353E-17
ENSSSCG00000021536	CLDN9	-2,02	1,641E-03
ENSSSCG00000038929	CEMIP	-2,01	1,018E-06
ENSSSCG00000016943	ADAMTS6	-2,00	5,181E-15
ENSSSCG00000023187	NA	-2,00	1,603E-03
ENSSSCG00000022230	CD9	-2,00	4,356E-26
ENSSSCG00000015780	STOX2	-2,00	7,471E-12
ENSSSCG00000010949	DAPK1	-2,00	9,349E-15
ENSSSCG00000013886	B3GNT3	-2,00	4,589E-27
ENSSSCG00000010012	SLC35E4	-1,99	3,807E-16
ENSSSCG00000001470	SLA-DMA	-1,99	2,541E-09
ENSSSCG00000005375	CORO2A	-1,99	7,583E-17
ENSSSCG00000035958	EVA1A	-1,98	1,207E-25
ENSSSCG00000010011	TCN2	-1,97	5,916E-31
ENSSSCG00000002383	FOS	-1,96	2,334E-06
ENSSSCG00000033136	NA	-1,96	2,374E-03
ENSSSCG00000013940	NLRP3	-1,95	3,981E-06
ENSSSCG00000040184	LMO7	-1,95	3,475E-18
ENSSSCG00000013292	PRR5L	-1,94	6,715E-15
ENSSSCG00000017868	NA	-1,94	2,441E-17
ENSSSCG00000015955	ITGA6	-1,93	9,131E-22
ENSSSCG00000040366	ADAMTSL1	-1,93	1,069E-08

ENSSSCG00000028047	DTNA	-1,93	3,605E-18
ENSSSCG00000039412	DOC2B	-1,93	1,888E-12
ENSSSCG00000022351	GSTO1	-1,92	2,948E-20
ENSSSCG00000013653	ICAM5	-1,92	4,671E-11
ENSSSCG00000036907	NMB	-1,92	1,348E-04
ENSSSCG00000038730	ITPRID2	-1,91	8,032E-15
ENSSSCG00000009833	SH2B3	-1,91	9,852E-29
ENSSSCG00000038948	ETS2	-1,91	7,165E-30
ENSSSCG00000029230	ECM1	-1,89	1,532E-14
ENSSSCG00000016725	TNS3	-1,89	1,283E-18
ENSSSCG00000011499	LRIG1	-1,88	2,120E-10
ENSSSCG00000011404	HYAL1	-1,87	1,620E-21
ENSSSCG00000023662	CHST3	-1,87	1,249E-29
ENSSSCG00000036905	NA	-1,87	5,223E-04
ENSSSCG00000011440	SEMA3G	-1,86	3,903E-15
ENSSSCG00000016450	NOS3	-1,85	6,450E-16
ENSSSCG00000006735	PTGFRN	-1,84	4,453E-07
ENSSSCG00000013632	C19orf38	-1,83	3,077E-04
ENSSSCG00000013654	NA	-1,82	7,424E-06
ENSSSCG00000009625	NA	-1,82	1,745E-15
ENSSSCG00000001469	SLA-DMB	-1,82	8,119E-07
ENSSSCG00000010122	CDC45	-1,82	7,174E-18
ENSSSCG00000006337	HSD17B7	-1,82	1,596E-22
ENSSSCG00000040942	NA	-1,81	8,412E-20
ENSSSCG00000021597	PHLDA2	-1,81	1,644E-17
ENSSSCG00000030655	MAMDC2	-1,81	1,770E-25
ENSSSCG00000016872	HMGCS1	-1,81	1,601E-22
ENSSSCG00000007189	SDCBP2	-1,81	3,443E-22
ENSSSCG00000012277	TIMP1	-1,79	7,223E-17
ENSSSCG00000036639	STOM	-1,79	8,904E-23
ENSSSCG00000013418	CFD	-1,77	2,274E-05
ENSSSCG00000036549	DPYSL3	-1,77	2,960E-25
ENSSSCG00000006577	S100A5	-1,75	6,094E-10
ENSSSCG00000008147	FHL2	-1,74	3,565E-19
ENSSSCG00000035715	GCH1	-1,74	3,475E-18
ENSSSCG00000037697	MGP	-1,73	4,246E-07
ENSSSCG00000016991	DUSP1	-1,72	1,752E-19
ENSSSCG00000010600	CALHM2	-1,72	3,602E-15
ENSSSCG00000012828	STARD8	-1,72	9,531E-20
ENSSSCG00000030269	NA	-1,72	3,658E-09
ENSSSCG00000040565	NA	-1,72	1,782E-03
ENSSSCG00000010746	ADAM12	-1,71	9,546E-13

ENSSSCG00000032980	NA	-1,71	4,980E-06
ENSSSCG00000038037	NA	-1,70	5,097E-04
ENSSSCG00000008510	LTBP1	-1,70	1,166E-17
ENSSSCG00000029813	TSPAN5	-1,69	4,892E-14
ENSSSCG00000002039	MMP14	-1,69	1,038E-15
ENSSSCG00000022998	PKIG	-1,69	2,679E-21
ENSSSCG00000007151	CDC25B	-1,69	3,154E-19
ENSSSCG00000029165	DOK4	-1,69	2,024E-16
ENSSSCG00000000423	LRP1	-1,68	4,481E-21
ENSSSCG00000040207	P2RY2	-1,68	4,137E-12
ENSSSCG00000004598	NA	-1,68	2,656E-17
ENSSSCG00000039426	FSTL1	-1,67	3,855E-10
ENSSSCG00000028048	ARHGEF15	-1,66	1,540E-07
ENSSSCG00000009778	RILPL2	-1,66	1,591E-21
ENSSSCG00000008898	NA	-1,66	8,585E-20
ENSSSCG00000011721	P2RY1	-1,65	2,295E-11
ENSSSCG00000016032	TFPI	-1,65	4,245E-12
ENSSSCG00000002263	SLCO3A1	-1,65	1,968E-06
ENSSSCG00000005376	TBC1D2	-1,65	1,356E-09
ENSSSCG00000011701	TM4SF18	-1,65	7,019E-05
ENSSSCG00000006612	S100A10	-1,65	8,273E-16
ENSSSCG00000002828	LPCAT2	-1,64	9,053E-20
ENSSSCG00000005203	IL33	-1,63	3,496E-14
ENSSSCG00000022247	PROSER2	-1,63	2,429E-15
ENSSSCG00000005701	ASS1	-1,62	6,408E-17
ENSSSCG00000008511	NA	-1,62	7,321E-04
ENSSSCG00000031888	DDIT4	-1,61	2,595E-23
ENSSSCG00000028512	LDLR	-1,61	1,934E-15
ENSSSCG00000033337	ARHGDIB	-1,61	5,570E-09
ENSSSCG00000033703	FAM111A	-1,60	1,028E-22
ENSSSCG00000021084	S100A6	-1,59	6,221E-10
ENSSSCG00000012634	DOCK11	-1,59	3,759E-11
ENSSSCG00000038506	NA	-1,58	1,349E-14
ENSSSCG00000005967	FAM84B	-1,57	4,388E-06
ENSSSCG00000026940	CASP10	-1,57	6,548E-22
ENSSSCG00000000370	DGKA	-1,57	1,483E-14
ENSSSCG00000031616	FOSB	-1,56	3,758E-02
ENSSSCG00000036768	PRAG1	-1,56	2,027E-15
ENSSSCG00000021306	GJC1	-1,56	8,553E-16
ENSSSCG00000012996	CDC42EP2	-1,55	7,906E-07
ENSSSCG00000004755	DLL4	-1,54	4,599E-17
ENSSSCG00000038500	TRIB1	-1,54	1,682E-13

ENSSSCG00000035420	HES4	-1,53	3,829E-17
ENSSSCG00000005688	PTGES	-1,53	1,641E-07
ENSSSCG00000036274	NA	-1,53	2,259E-11
ENSSSCG00000036886	ZNF792	-1,53	6,452E-07
ENSSSCG00000005094	TMEM30B	-1,53	5,510E-06
ENSSSCG00000008601	SDC1	-1,53	1,080E-16
ENSSSCG00000015083	FXYD6	-1,52	3,947E-18
ENSSSCG00000031866	TIMP3	-1,52	9,776E-09
ENSSSCG00000001917	CD276	-1,50	3,228E-16
ENSSSCG00000006742	MAB21L3	-1,50	3,924E-13
ENSSSCG00000034510	ORAI1	-1,50	2,312E-17
ENSSSCG00000030271	GSTO2	-1,50	5,924E-04
ENSSSCG00000015285	LRRN2	-1,49	3,445E-03
ENSSSCG00000010239	RUFY2	-1,49	3,543E-03
ENSSSCG00000040507	PDLIM7	-1,49	1,852E-15
ENSSSCG00000006002	CCN3	-1,49	2,901E-19
ENSSSCG00000023956	NEDD9	-1,49	2,585E-17
ENSSSCG00000034863	PARD6A	-1,48	2,974E-04
ENSSSCG00000004668	SLC30A4	-1,48	5,162E-14
ENSSSCG00000034739	NA	-1,48	2,142E-12
ENSSSCG00000003079	NA	-1,48	1,262E-14
ENSSSCG00000023273	SH3YL1	-1,48	5,782E-05
ENSSSCG00000003707	NPC1	-1,48	1,023E-15
ENSSSCG00000038536	GPR158	-1,47	2,452E-08
ENSSSCG00000031392	NA	-1,47	1,294E-05
ENSSSCG00000022345	NA	-1,47	1,720E-13
ENSSSCG00000010151	LYST	-1,46	1,281E-11
ENSSSCG00000025208	RNF39	-1,45	1,738E-05
ENSSSCG00000017052	ADAM19	-1,45	3,219E-09
ENSSSCG00000006687	ITGA10	-1,45	9,390E-11
ENSSSCG00000002259	NA	-1,45	1,935E-18
ENSSSCG00000038598	ADRB2	-1,44	2,377E-07
ENSSSCG00000015277	SOX13	-1,44	1,140E-16
ENSSSCG00000008930	TMPRSS11F	-1,44	5,637E-14
ENSSSCG00000031074	FAM110D	-1,44	1,455E-12
ENSSSCG00000033423	NA	-1,43	5,850E-13
ENSSSCG00000001639	TRERF1	-1,43	1,928E-11
ENSSSCG00000035403	RFX2	-1,43	8,801E-06
ENSSSCG00000035027	ZDHHC14	-1,43	3,996E-13
ENSSSCG00000032761	LAMA5	-1,43	3,180E-09
ENSSSCG00000032956	C16orf45	-1,42	2,780E-04
ENSSSCG00000016912	PLPP1	-1,42	1,164E-17

ENSSSCG00000030325	C1QTNF6	-1,41	3,614E-11
ENSSSCG00000011209	NA	-1,41	6,371E-10
ENSSSCG00000014395	PCDH12	-1,40	2,742E-08
ENSSSCG00000005344	MELK	-1,40	1,935E-09
ENSSSCG00000006296	ATP1B1	-1,39	1,215E-10
ENSSSCG00000016851	OSMR	-1,38	1,957E-14
ENSSSCG00000031509	MTMR12	-1,38	5,709E-14
ENSSSCG00000009320	FLT1	-1,38	1,907E-16
ENSSSCG00000005620	SH2D3C	-1,38	2,744E-12
ENSSSCG00000010035	YWHAH	-1,38	4,824E-16
ENSSSCG00000010613	ITPRIP	-1,38	4,015E-15
ENSSSCG00000006289	F5	-1,38	1,810E-13
ENSSSCG00000009377	THSD1	-1,38	2,011E-08
ENSSSCG00000011666	CLSTN2	-1,37	2,448E-02
ENSSSCG00000006893	BCAR3	-1,36	1,781E-11
ENSSSCG00000033202	NA	-1,36	9,777E-03
ENSSSCG00000001727	TNFRSF21	-1,36	4,461E-15
ENSSSCG00000008103	MERTK	-1,36	1,299E-10
ENSSSCG00000010980	C9orf24	-1,36	3,473E-03
ENSSSCG00000023639	NRM	-1,36	1,062E-09
ENSSSCG00000013307	LMO2	-1,35	3,245E-13
ENSSSCG00000034259	PMEPA1	-1,35	4,673E-17
ENSSSCG00000011074	ARHGAP21	-1,35	1,193E-09
ENSSSCG00000036132	CKM	-1,35	1,035E-08
ENSSSCG00000039998	CLIC2	-1,35	1,396E-12
ENSSSCG00000027778	NA	-1,35	1,103E-15
ENSSSCG00000026454	PMAIP1	-1,34	6,993E-10
ENSSSCG00000007872	XYLT1	-1,34	5,963E-08
ENSSSCG00000007149	SPEF1	-1,33	6,655E-05
ENSSSCG00000001918	NPTN	-1,33	6,209E-15
ENSSSCG00000017993	NTN1	-1,33	2,459E-08
ENSSSCG00000002754	NQO1	-1,32	2,216E-11
ENSSSCG00000031174	FBP1	-1,32	1,550E-04
ENSSSCG00000009896	BICDL1	-1,32	4,057E-03
ENSSSCG00000006335	RGS4	-1,32	2,855E-10
ENSSSCG00000003108	NPAS1	-1,31	5,403E-04
ENSSSCG00000032241	GPNMB	-1,31	2,840E-15
ENSSSCG00000011264	CSRNP1	-1,31	3,408E-11
ENSSSCG00000009815	CAMKK2	-1,31	3,950E-11
ENSSSCG00000011570	IRAK2	-1,31	2,466E-11
ENSSSCG00000001076	RNF144B	-1,30	7,215E-08
ENSSSCG00000040617	TNFAIP8	-1,30	7,026E-04

ENSSSCG00000023611	NA	-1,30	3,348E-13
ENSSSCG00000003458	EFHD2	-1,30	1,836E-11
ENSSSCG00000027480	KLF10	-1,30	1,224E-13
ENSSSCG00000022173	ANKRD50	-1,30	1,553E-15
ENSSSCG00000008434	TTC7A	-1,30	9,794E-09
ENSSSCG00000008240	NA	-1,30	1,480E-02
ENSSSCG00000005944	NDRG1	-1,29	5,689E-14
ENSSSCG00000015022	LAYN	-1,29	4,857E-05
ENSSSCG00000010772	ADAM8	-1,29	2,066E-04
ENSSSCG00000011951	NFKBIZ	-1,29	3,565E-08
ENSSSCG00000013366	NA	-1,28	1,681E-10
ENSSSCG00000005305	UNC13B	-1,28	6,120E-14
ENSSSCG00000009083	SPRY1	-1,28	5,508E-08
ENSSSCG00000006970	DLC1	-1,27	7,534E-12
ENSSSCG00000031053	S100A1	-1,27	7,318E-04
ENSSSCG00000029066	NA	-1,27	4,550E-14
ENSSSCG00000008844	KDR	-1,27	4,289E-07
ENSSSCG00000040618	NA	-1,27	4,784E-02
ENSSSCG00000016687	NA	-1,27	8,088E-12
ENSSSCG00000005030	NID2	-1,26	7,829E-07
ENSSSCG00000032176	SMURF2	-1,26	4,656E-12
ENSSSCG00000003909	PIK3R3	-1,26	3,976E-12
ENSSSCG00000028889	VPS37B	-1,26	3,759E-11
ENSSSCG00000001930	PKM	-1,26	1,818E-06
ENSSSCG00000012309	SHROOM4	-1,25	3,408E-11
ENSSSCG00000030831	DENND3	-1,25	1,015E-09
ENSSSCG00000010683	GRK5	-1,25	9,319E-11
ENSSSCG00000037748	NA	-1,25	1,123E-07
ENSSSCG00000002688	PLCG2	-1,24	3,926E-13
ENSSSCG00000036236	ELOVL6	-1,24	1,159E-11
ENSSSCG00000038080	EMCN	-1,24	1,733E-07
ENSSSCG00000011859	HEG1	-1,24	1,294E-06
ENSSSCG00000032591	NA	-1,24	6,489E-11
ENSSSCG00000000804	ANO6	-1,24	1,435E-10
ENSSSCG00000032525	NA	-1,23	2,692E-03
ENSSSCG00000031579	PCP4L1	-1,23	1,860E-02
ENSSSCG00000039060	RASSF3	-1,23	3,543E-11
ENSSSCG00000016932	DEPDC1B	-1,23	2,338E-05
ENSSSCG00000005078	DAAM1	-1,22	4,618E-12
ENSSSCG00000004138	HIVEP2	-1,22	2,564E-10
ENSSSCG00000022288	STK38L	-1,21	4,178E-14
ENSSSCG00000014255	SLC12A2	-1,21	6,586E-07

ENSSSCG00000012490	TMEM35A	-1,21	5,477E-07
ENSSSCG00000000293	ITGA5	-1,21	7,607E-06
ENSSSCG00000013397	ARNTL	-1,21	3,974E-08
ENSSSCG00000030278	MLLT11	-1,20	2,376E-10
ENSSSCG00000023377	EMP3	-1,20	4,404E-11
ENSSSCG00000033695	KCTD15	-1,20	7,343E-11
ENSSSCG00000040578	CASKIN2	-1,20	1,526E-11
ENSSSCG00000015579	PTGS2	-1,20	2,823E-12
ENSSSCG00000014570	NRIP3	-1,20	2,327E-03
ENSSSCG00000033410	INKA1	-1,19	5,113E-08
ENSSSCG00000037209	TLNRD1	-1,19	8,952E-13
ENSSSCG00000004058	EZR	-1,19	3,898E-11
ENSSSCG00000036437	NOG	-1,19	7,718E-06
ENSSSCG00000014156	ARRDC3	-1,19	8,104E-12
ENSSSCG00000022933	EFR3B	-1,19	6,744E-08
ENSSSCG00000023128	NA	-1,19	1,625E-02
ENSSSCG00000032768	NA	-1,19	1,258E-12
ENSSSCG00000014965	ENDOD1	-1,18	5,520E-09
ENSSSCG00000037483	RFTN2	-1,18	5,280E-11
ENSSSCG00000020912	HECTD2	-1,18	2,663E-06
ENSSSCG00000030882	NA	-1,18	1,485E-02
ENSSSCG00000008239	CAPG	-1,18	6,014E-08
ENSSSCG00000033521	NA	-1,17	1,394E-03
ENSSSCG00000011330	NBEAL2	-1,17	4,546E-10
ENSSSCG00000000807	SLC38A1	-1,17	1,966E-03
ENSSSCG00000009348	STARD13	-1,17	6,066E-07
ENSSSCG00000025114	FMNL3	-1,17	3,619E-12
ENSSSCG00000007554	ZFAND2A	-1,17	3,378E-11
ENSSSCG00000017956	CD68	-1,16	1,562E-07
ENSSSCG00000017232	SLC9A3R1	-1,16	6,561E-07
ENSSSCG00000013457	DOT1L	-1,16	1,364E-09
ENSSSCG00000012141	CA5B	-1,16	4,222E-07
ENSSSCG00000008723	HTRA3	-1,15	5,533E-06
ENSSSCG00000011437	ALAS1	-1,15	3,497E-07
ENSSSCG00000000656	CLEC2B	-1,15	3,441E-11
ENSSSCG00000017723	CCL2	-1,15	2,107E-03
ENSSSCG00000016658	ANLN	-1,15	9,674E-07
ENSSSCG00000036451	NA	-1,14	2,258E-05
ENSSSCG00000012986	KCNK7	-1,14	1,217E-02
ENSSSCG00000026116	FHOD1	-1,14	7,255E-11
ENSSSCG00000007286	ACSS2	-1,14	1,570E-11
ENSSSCG00000008631	PQLC3	-1,14	3,347E-11

ENSSSCG00000035568	NA	-1,13	8,486E-03
ENSSSCG00000013768	MISP3	-1,13	2,156E-08
ENSSSCG00000003848	LRP8	-1,13	2,819E-08
ENSSSCG00000016853	RICTOR	-1,12	6,766E-10
ENSSSCG00000021161	CKS2	-1,12	9,604E-08
ENSSSCG00000025499	NA	-1,12	2,787E-09
ENSSSCG00000004454	ME1	-1,12	4,367E-11
ENSSSCG00000002788	EXOC3L1	-1,12	3,180E-08
ENSSSCG00000021359	CDC42EP3	-1,12	1,757E-10
ENSSSCG00000009755	AACS	-1,12	4,257E-12
ENSSSCG00000017513	NPEPPS	-1,12	5,963E-11
ENSSSCG00000038423	GLA	-1,11	1,734E-09
ENSSSCG00000000699	LPAR5	-1,11	1,230E-02
ENSSSCG00000009122	ARSJ	-1,11	2,238E-09
ENSSSCG00000007115	THBD	-1,11	1,052E-04
ENSSSCG00000036488	KLF3	-1,10	1,208E-11
ENSSSCG00000014080	HMGCR	-1,10	1,461E-10
ENSSSCG00000017879	NA	-1,10	9,519E-09
ENSSSCG00000023235	MAN1C1	-1,10	4,142E-03
ENSSSCG00000037184	GPR4	-1,10	8,417E-05
ENSSSCG00000032489	KLHDC1	-1,09	1,659E-03
ENSSSCG00000035355	F2R	-1,09	2,051E-05
ENSSSCG00000013086	CYB561A3	-1,09	2,135E-07
ENSSSCG00000026984	DIPK2B	-1,09	1,986E-04
ENSSSCG00000026689	NA	-1,09	9,218E-03
ENSSSCG00000001233	TRIM26	-1,09	1,263E-10
ENSSSCG00000013410	SWAP70	-1,08	2,394E-09
ENSSSCG00000010085	SDF2L1	-1,08	1,436E-05
ENSSSCG00000030359	ARHGEF3	-1,08	2,996E-06
ENSSSCG00000003569	SLC9A1	-1,08	8,203E-10
ENSSSCG00000013933	PBX4	-1,08	4,607E-03
ENSSSCG00000004191	MOXD1	-1,07	9,863E-03
ENSSSCG00000004412	MICAL1	-1,07	3,813E-08
ENSSSCG00000036705	RPH3AL	-1,07	1,886E-06
ENSSSCG00000015537	XPR1	-1,07	8,698E-08
ENSSSCG00000031979	NA	-1,07	4,718E-04
ENSSSCG00000005970	SQLE	-1,07	6,380E-11
ENSSSCG00000035455	FAM219A	-1,07	9,219E-11
ENSSSCG00000007188	NA	-1,06	1,133E-06
ENSSSCG00000015496	CENPL	-1,06	1,351E-03
ENSSSCG00000004806	NA	-1,06	5,625E-06
ENSSSCG00000040332	LBH	-1,06	1,982E-06

ENSSSCG00000006550	ATP8B2	-1,06	4,126E-09
ENSSSCG00000004049	ACAT2	-1,06	3,412E-10
ENSSSCG00000036946	NA	-1,05	1,168E-02
ENSSSCG00000011538	LMCD1	-1,05	1,307E-04
ENSSSCG00000033120	PALM2	-1,05	1,152E-06
ENSSSCG00000014113	HOMER1	-1,05	5,961E-10
ENSSSCG00000004825	CHSY1	-1,05	5,109E-10
ENSSSCG00000038494	LPIN2	-1,05	3,517E-07
ENSSSCG00000026233	NA	-1,05	3,045E-05
ENSSSCG00000032330	THY1	-1,05	1,034E-06
ENSSSCG00000011194	ANKRD28	-1,04	3,046E-09
ENSSSCG00000023267	SCN1B	-1,04	1,533E-02
ENSSSCG00000032728	EFNB1	-1,04	4,139E-06
ENSSSCG00000004421	FYN	-1,04	1,445E-10
ENSSSCG00000011333	NA	-1,04	8,546E-03
ENSSSCG00000040815	DUSP5	-1,04	1,324E-04
ENSSSCG00000029391	PLSCR3	-1,04	2,422E-07
ENSSSCG00000032399	NA	-1,04	1,001E-07
ENSSSCG00000002716	MLKL	-1,04	1,755E-07
ENSSSCG00000021053	GIPR	-1,04	1,166E-03
ENSSSCG00000038843	ST3GAL2	-1,03	2,033E-09
ENSSSCG00000035941	DSEL	-1,03	5,113E-08
ENSSSCG00000037468	GNE	-1,03	7,454E-07
ENSSSCG00000022649	SLC7A11	-1,03	3,124E-07
ENSSSCG00000022162	RAB11FIP5	-1,03	4,277E-08
ENSSSCG00000021712	HERC6	-1,03	1,603E-09
ENSSSCG00000034133	NA	-1,02	3,505E-02
ENSSSCG00000037621	KIAA1522	-1,02	1,387E-06
ENSSSCG00000022777	SLC35C2	-1,02	7,124E-07
ENSSSCG00000010608	SFR1	-1,02	1,166E-02
ENSSSCG00000015595	ATF3	-1,02	1,550E-04
ENSSSCG00000039071	NA	-1,02	1,463E-06
ENSSSCG00000026326	CCNF	-1,01	9,958E-05
ENSSSCG00000032613	SNAI1	-1,01	2,509E-05
ENSSSCG00000013655	ICAM1	-1,01	1,030E-06
ENSSSCG00000007157	SLC4A11	-1,01	1,696E-09
ENSSSCG00000023526	RAPGEF3	-1,01	5,605E-08
ENSSSCG00000025447	MID1IP1	-1,01	1,789E-06
ENSSSCG00000029326	CCNB1	-1,01	3,581E-05
ENSSSCG00000011498	SLC25A26	-1,01	2,852E-06
ENSSSCG00000011430	DUSP7	-1,00	3,497E-07
ENSSSCG00000005240	DOCK8	-1,00	4,184E-06

ENSSSCG00000025924	IGFBP5	8,44	2,219E-154
ENSSSCG00000017607	TMEM100	7,29	2,150E-19
ENSSSCG00000040266	MDFIC2	6,36	5,791E-98
ENSSSCG00000012002	ROBO2	6,29	2,598E-23
ENSSSCG00000012003	NA	6,24	1,586E-08
ENSSSCG00000034943	GDF6	4,87	1,204E-23
ENSSSCG00000007730	CALN1	4,85	1,399E-08
ENSSSCG00000014978	ANGPTL5	4,84	1,328E-22
ENSSSCG00000003439	DHRS3	4,76	2,931E-24
ENSSSCG00000014011	RASGEF1C	4,61	1,005E-17
ENSSSCG00000032474	CXCL10	4,60	1,652E-06
ENSSSCG00000007486	CYP24A1	4,49	8,491E-34
ENSSSCG00000025992	ENPP3	4,39	7,402E-14
ENSSSCG00000009111	SYNPO2	4,33	9,939E-58
ENSSSCG00000005393	PLPPR1	4,22	1,239E-28
ENSSSCG00000032601	RASSF2	4,08	1,639E-35
ENSSSCG00000008284	NA	4,05	5,122E-10
ENSSSCG00000009456	KLHL1	3,99	1,229E-18
ENSSSCG00000038783	IGFBP3	3,95	2,385E-16
ENSSSCG00000032878	SHISA9	3,92	1,967E-17
ENSSSCG00000032423	SPOCK1	3,91	1,671E-27
ENSSSCG00000009125	ANK2	3,89	2,119E-22
ENSSSCG00000029710	GCNT4	3,79	3,872E-25
ENSSSCG00000028931	GDF7	3,77	1,295E-50
ENSSSCG00000006161	IL7	3,77	2,421E-45
ENSSSCG00000040648	CCL11	3,67	1,440E-09
ENSSSCG00000007678	COL26A1	3,67	5,223E-14
ENSSSCG00000003753	NA	3,67	8,914E-08
ENSSSCG00000007073	ISM1	3,65	1,022E-31
ENSSSCG00000011195	GALNT15	3,62	1,467E-92
ENSSSCG00000013599	ANGPTL4	3,59	8,722E-36
ENSSSCG00000039821	GPRIN3	3,58	1,224E-25
ENSSSCG00000001944	NKX2-8	3,58	4,541E-33
ENSSSCG00000015334	PDK4	3,55	6,290E-90
ENSSSCG00000031988	MYOCD	3,52	1,350E-07
ENSSSCG00000028911	COLQ	3,52	1,031E-10
ENSSSCG00000036742	KLF15	3,52	2,687E-25
ENSSSCG00000004157	IL20RA	3,46	1,044E-08
ENSSSCG00000003709	LAMA3	3,43	1,173E-16
ENSSSCG00000009720	DDX60	3,32	1,382E-06
ENSSSCG00000005045	BMP4	3,22	2,772E-37
ENSSSCG00000011129	ITIH5	3,22	4,708E-25

ENSSSCG00000040903	NA	3,20	4,791E-05
ENSSSCG00000033043	SHANK2	3,18	6,465E-09
ENSSSCG00000009222	SPARCL1	3,16	9,633E-19
ENSSSCG00000013408	ADM	3,15	1,561E-58
ENSSSCG00000007391	MATN4	3,15	4,012E-13
ENSSSCG00000014310	CXCL14	3,13	1,405E-39
ENSSSCG00000011110	CCDC3	3,13	2,424E-09
ENSSSCG00000026602	PTGIR	3,12	1,824E-12
ENSSSCG00000037890	ZNF853	3,12	1,223E-12
ENSSSCG00000028549	ECM2	3,09	8,707E-16
ENSSSCG00000006321	FAM78B	3,04	5,436E-22
ENSSSCG00000001723	PLA2G7	3,04	6,486E-12
ENSSSCG00000035720	HRCT1	3,02	2,492E-22
ENSSSCG00000015523	RALGPS2	3,01	2,419E-58
ENSSSCG00000004017	FRMD1	2,93	1,453E-10
ENSSSCG00000005268	RORB	2,91	4,410E-12
ENSSSCG00000035598	EDN1	2,91	8,490E-21
ENSSSCG00000000663	NA	2,90	1,872E-09
ENSSSCG00000036201	NPR3	2,89	6,485E-21
ENSSSCG00000002314	SMOC1	2,86	2,911E-42
ENSSSCG00000016613	AASS	2,86	7,234E-12
ENSSSCG00000039587	NA	2,82	5,389E-24
ENSSSCG00000023924	PDE1A	2,81	3,036E-19
ENSSSCG00000014900	RAB30	2,71	4,605E-14
ENSSSCG00000012510	ARMCX2	2,70	8,755E-26
ENSSSCG00000008604	WDR35	2,68	8,083E-13
ENSSSCG00000008397	EFEMP1	2,67	1,333E-03
ENSSSCG00000010222	ZNF365	2,63	2,255E-08
ENSSSCG00000033463	KCNF1	2,62	1,061E-04
ENSSSCG00000001716	RCAN2	2,59	4,461E-15
ENSSSCG00000029388	PDE2A	2,58	6,446E-23
ENSSSCG00000008230	ATOH8	2,56	4,226E-58
ENSSSCG00000038866	NA	2,56	8,583E-05
ENSSSCG00000015753	ANGPT2	2,56	1,613E-15
ENSSSCG00000006624	SELENBP1	2,55	1,666E-12
ENSSSCG00000013333	BDNF	2,51	5,916E-20
ENSSSCG00000002852	PLEKHF1	2,50	4,525E-36
ENSSSCG00000023771	NA	2,49	9,317E-12
ENSSSCG00000038358	NA	2,48	2,169E-02
ENSSSCG00000006051	CTHRC1	2,47	7,458E-20
ENSSSCG00000011212	RARB	2,45	4,783E-15
ENSSSCG00000032936	PIM3	2,45	5,154E-31

ENSSSCG00000017131	FN3K	2,45	2,418E-09
ENSSSCG00000036622	NA	2,44	2,446E-05
ENSSSCG00000027762	TNFRSF11B	2,44	4,379E-13
ENSSSCG00000014979	CEP126	2,44	1,989E-12
ENSSSCG00000031856	DACT1	2,44	1,506E-12
ENSSSCG00000004572	NA	2,44	3,802E-33
ENSSSCG00000039983	GJA4	2,41	1,552E-20
ENSSSCG00000000500	NA	2,41	7,260E-13
ENSSSCG00000030309	NDRG2	2,40	1,623E-23
ENSSSCG00000039613	SVIP	2,39	2,855E-10
ENSSSCG00000010212	NA	2,39	7,445E-10
ENSSSCG00000037241	RGS2	2,39	1,057E-33
ENSSSCG00000008618	MYCN	2,38	1,487E-08
ENSSSCG00000027312	PTCH1	2,37	3,566E-16
ENSSSCG00000000915	DCN	2,37	1,445E-06
ENSSSCG00000036360	LURAP1L	2,37	1,814E-11
ENSSSCG00000011874	PARP14	2,36	2,518E-07
ENSSSCG00000025967	SMKR1	2,36	9,648E-06
ENSSSCG00000032398	VGLL3	2,34	8,465E-25
ENSSSCG00000011495	PRICKLE2	2,33	1,198E-28
ENSSSCG00000013664	C19orf66	2,33	2,540E-07
ENSSSCG00000011979	GPR15	2,32	2,786E-19
ENSSSCG00000018007	MYH3	2,31	1,065E-08
ENSSSCG00000034763	IRS2	2,29	4,243E-11
ENSSSCG00000006719	NA	2,28	5,721E-07
ENSSSCG00000029201	AJUBA	2,27	1,484E-24
ENSSSCG00000012679	GPC4	2,26	5,174E-10
ENSSSCG00000035621	GABRB1	2,26	5,629E-10
ENSSSCG00000003881	SPATA6	2,25	1,912E-17
ENSSSCG00000007061	PAK5	2,25	4,806E-08
ENSSSCG00000002637	NA	2,25	2,833E-04
ENSSSCG00000016225	MOGAT1	2,24	6,962E-12
ENSSSCG00000007564	AMZ1	2,23	6,365E-08
ENSSSCG00000009240	PLAC8	2,23	5,625E-06
ENSSSCG00000034213	ACER2	2,22	3,506E-24
ENSSSCG00000004570	TPM1	2,20	4,280E-11
ENSSSCG00000004540	ONECUT2	2,20	1,196E-05
ENSSSCG00000038345	IKZF1	2,20	2,318E-05
ENSSSCG00000035987	EHD3	2,19	1,660E-26
ENSSSCG00000001090	ALDH5A1	2,19	3,550E-09
ENSSSCG00000016983	STC2	2,18	6,953E-17
ENSSSCG00000009537	NA	2,18	1,199E-04

ENSSSCG00000030515	GABRA4	2,17	5,018E-16
ENSSSCG00000025416	CAMKV	2,17	3,615E-24
ENSSSCG00000010219	ARID5B	2,16	2,146E-29
ENSSSCG00000031524	NA	2,16	8,930E-12
ENSSSCG00000001914	LOXL1	2,16	1,159E-23
ENSSSCG00000009281	SGCG	2,14	1,074E-03
ENSSSCG00000035595	HMCN1	2,12	9,310E-27
ENSSSCG00000036831	EFNA4	2,12	5,276E-06
ENSSSCG00000033508	NA	2,12	4,142E-03
ENSSSCG00000039646	NA	2,10	9,058E-04
ENSSSCG00000015839	NRG1	2,09	6,591E-22
ENSSSCG00000004844	MTMR10	2,09	1,406E-26
ENSSSCG00000010451	IFIT2	2,09	6,920E-10
ENSSSCG00000029783	MKX	2,09	1,795E-14
ENSSSCG00000024973	NA	2,08	6,480E-12
ENSSSCG00000027157	SLC40A1	2,07	2,197E-24
ENSSSCG00000013393	SPON1	2,07	1,278E-16
ENSSSCG00000012780	PLXNB3	2,07	6,715E-15
ENSSSCG00000022630	FLYWCH2	2,06	6,004E-07
ENSSSCG00000032251	MAFB	2,06	1,243E-06
ENSSSCG00000014141	RFESD	2,06	9,068E-36
ENSSSCG00000029796	KBTBD11	2,05	3,334E-06
ENSSSCG00000006874	PALMD	2,05	2,686E-26
ENSSSCG00000004433	HS3ST5	2,05	1,245E-23
ENSSSCG00000004898	TNFRSF11A	2,04	2,504E-09
ENSSSCG00000025286	MCTP1	2,04	2,079E-07
ENSSSCG00000028996	ALDH1A1	2,04	7,007E-29
ENSSSCG00000021460	NYX	2,04	5,733E-05
ENSSSCG00000016885	ITGA1	2,04	4,199E-13
ENSSSCG00000014316	TGFBI	2,04	5,589E-22
ENSSSCG00000009676	ZNF395	2,04	7,686E-29
ENSSSCG00000031960	NA	2,03	3,667E-11
ENSSSCG00000036049	PEG10	2,03	2,327E-15
ENSSSCG00000004192	CCN2	2,02	8,132E-11
ENSSSCG00000039707	RTKN2	2,02	3,132E-22
ENSSSCG00000009114	PRSS12	2,01	1,350E-15
ENSSSCG00000033089	NA	2,01	1,445E-06
ENSSSCG00000014140	GPR150	2,01	4,838E-27
ENSSSCG00000021208	SELENOP	2,00	1,077E-26
ENSSSCG00000017255	ABCA5	2,00	1,053E-06
ENSSSCG00000008787	KLHL5	2,00	2,228E-32
ENSSSCG00000037318	TRABD2B	1,99	1,568E-20

ENSSSCG00000024537	CYP2C42	1,99	3,856E-09
ENSSSCG00000011176	SUSD4	1,99	6,120E-05
ENSSSCG00000036155	FAT4	1,99	9,520E-30
ENSSSCG00000010054	ADORA2A	1,99	3,100E-14
ENSSSCG00000028814	SOD3	1,99	3,596E-22
ENSSSCG00000011980	CLDND1	1,99	2,271E-21
ENSSSCG00000000062	CSDC2	1,98	3,441E-11
ENSSSCG00000016034	COL3A1	1,98	1,117E-18
ENSSSCG00000017296	ACE	1,98	3,038E-11
ENSSSCG00000016784	ANKH	1,96	4,614E-29
ENSSSCG00000014976	ARHGAP42	1,96	2,715E-18
ENSSSCG00000040130	FAM155A	1,96	3,998E-05
ENSSSCG00000035284	BMF	1,96	2,555E-24
ENSSSCG00000000090	CBX7	1,95	1,776E-07
ENSSSCG00000024206	CPPED1	1,95	2,333E-18
ENSSSCG00000005486	KIF12	1,95	4,179E-12
ENSSSCG00000016589	LRRC4	1,93	2,663E-05
ENSSSCG00000040566	NA	1,93	2,016E-15
ENSSSCG00000004419	MFSD4B	1,93	8,066E-21
ENSSSCG00000016381	SNED1	1,92	6,865E-16
ENSSSCG00000029324	COLEC10	1,92	2,052E-06
ENSSSCG00000012941	SLC29A2	1,92	5,507E-05
ENSSSCG00000033001	FZD8	1,91	6,273E-13
ENSSSCG00000000478	GRIP1	1,91	5,350E-22
ENSSSCG00000023178	BATF2	1,91	1,053E-06
ENSSSCG00000015085	IL10RA	1,91	9,349E-15
ENSSSCG00000032181	C17orf58	1,91	1,827E-12
ENSSSCG00000034192	GNAO1	1,91	1,534E-11
ENSSSCG00000040498	FAM241B	1,89	2,759E-03
ENSSSCG00000032151	CPZ	1,89	1,772E-20
ENSSSCG00000011463	IL17RD	1,87	1,561E-06
ENSSSCG00000011583	TMEM40	1,87	3,066E-12
ENSSSCG00000021027	PGBD5	1,86	9,125E-05
ENSSSCG00000010452	IFIT1	1,86	3,725E-08
ENSSSCG00000023684	MT1A	1,86	2,544E-06
ENSSSCG00000008749	SLIT2	1,86	1,538E-05
ENSSSCG00000017416	DHX58	1,86	1,708E-08
ENSSSCG00000009361	POSTN	1,86	5,720E-09
ENSSSCG00000005338	RECK	1,86	1,654E-15
ENSSSCG00000011214	NGLY1	1,85	2,606E-27
ENSSSCG00000003733	KLHL14	1,84	4,388E-03
ENSSSCG00000038867	PPM1K	1,84	3,940E-21

ENSSSCG00000029754	SLC39A2	1,84	8,541E-03
ENSSSCG00000002997	NA	1,84	5,282E-04
ENSSSCG00000014975	NA	1,83	9,182E-08
ENSSSCG00000008926	TMPRSS11D	1,83	2,695E-04
ENSSSCG00000009937	ACACB	1,83	2,621E-23
ENSSSCG00000031176	GP9	1,83	4,851E-14
ENSSSCG00000023305	NA	1,83	2,162E-07
ENSSSCG00000003826	HOOK1	1,83	5,559E-12
ENSSSCG00000031733	AKAP12	1,82	3,697E-21
ENSSSCG00000008202	CNNM4	1,82	1,001E-10
ENSSSCG00000027901	CLDN22	1,82	2,426E-03
ENSSSCG00000030921	APOA1	1,82	1,813E-11
ENSSSCG00000006969	TRMT9B	1,81	2,771E-13
ENSSSCG00000011198	RFTN1	1,81	6,782E-19
ENSSSCG00000017601	TOM1L1	1,80	2,517E-19
ENSSSCG00000005284	GNA14	1,80	1,754E-10
ENSSSCG00000003744	MOCOS	1,80	3,020E-05
ENSSSCG00000014902	ANKRD42	1,79	1,951E-13
ENSSSCG00000000515	TRHDE	1,79	1,185E-06
ENSSSCG00000004875	CYB5A	1,78	3,125E-16
ENSSSCG00000039761	MYCL	1,78	6,923E-08
ENSSSCG00000007369	NA	1,78	5,240E-05
ENSSSCG00000010826	MARC2	1,78	2,302E-13
ENSSSCG00000003115	MEIS3	1,77	5,843E-11
ENSSSCG00000035077	INHBA	1,77	8,205E-09
ENSSSCG00000026453	ACSM5	1,76	2,584E-04
ENSSSCG00000021204	HOXA10	1,76	6,794E-14
ENSSSCG00000038966	KRT7	1,76	5,592E-05
ENSSSCG00000014672	NA	1,75	3,799E-11
ENSSSCG00000003805	PDE4B	1,74	2,294E-15
ENSSSCG00000029945	GCAT	1,74	6,824E-06
ENSSSCG00000029651	SLN	1,73	6,600E-05
ENSSSCG00000032667	NA	1,72	4,271E-04
ENSSSCG00000027870	NA	1,72	1,906E-02
ENSSSCG00000011806	MASP1	1,72	1,556E-08
ENSSSCG00000001042	MAK	1,72	6,573E-09
ENSSSCG00000004193	ENPP1	1,71	2,994E-21
ENSSSCG00000025126	LGI4	1,71	3,251E-06
ENSSSCG00000038774	MAPK13	1,71	9,042E-06
ENSSSCG00000011514	MITF	1,71	2,702E-11
ENSSSCG00000011412	CACNA2D2	1,70	3,808E-06
ENSSSCG00000022092	CYP7B1	1,70	9,592E-11

ENSSSCG00000033321	GAS1	1,69	4,097E-03
ENSSSCG00000002037	CDH24	1,69	7,693E-06
ENSSSCG00000034802	NA	1,69	2,850E-08
ENSSSCG00000040985	KCTD7	1,69	5,605E-08
ENSSSCG00000035511	C1QTNF1	1,69	1,592E-14
ENSSSCG00000015540	MR1	1,69	1,297E-10
ENSSSCG00000040445	RND3	1,68	1,080E-16
ENSSSCG00000025357	LDAH	1,68	3,327E-17
ENSSSCG00000024377	RPS6KA5	1,68	1,017E-12
ENSSSCG00000015045	NCAM1	1,68	1,519E-10
ENSSSCG00000037330	NA	1,68	7,486E-03
ENSSSCG00000022402	GRAMD2B	1,67	1,476E-12
ENSSSCG00000015413	FGL2	1,66	3,814E-05
ENSSSCG00000005277	GCNT1	1,65	4,106E-15
ENSSSCG00000036512	FGFRL1	1,65	1,694E-14
ENSSSCG00000009293	NA	1,64	5,641E-11
ENSSSCG00000026001	DNAJC1	1,64	5,916E-20
ENSSSCG00000011793	LIPH	1,64	8,091E-13
ENSSSCG00000005511	TRAF1	1,63	4,056E-08
ENSSSCG00000030303	ACHE	1,63	1,746E-04
ENSSSCG00000002764	SMPD3	1,62	5,502E-14
ENSSSCG00000012076	MX2	1,62	1,431E-05
ENSSSCG00000008902	PPAT	1,61	5,112E-12
ENSSSCG00000031831	CADM1	1,61	3,332E-16
ENSSSCG00000001715	ENPP5	1,61	5,117E-04
ENSSSCG00000016033	GULP1	1,61	2,352E-10
ENSSSCG00000038703	CCDC178	1,61	1,868E-04
ENSSSCG00000033327	PDGFB	1,61	6,888E-12
ENSSSCG00000010829	NA	1,60	7,797E-16
ENSSSCG00000009300	WASF3	1,60	5,253E-12
ENSSSCG00000000010	FBLN1	1,60	4,983E-10
ENSSSCG00000016863	OXCT1	1,59	3,741E-17
ENSSSCG00000011756	NLGN1	1,59	1,985E-06
ENSSSCG00000012504	NAP1L3	1,59	3,100E-14
ENSSSCG00000026583	TLR1	1,59	4,773E-05
ENSSSCG00000040946	EIF5A2	1,59	1,646E-12
ENSSSCG00000036383	LGALS3BP	1,59	2,662E-09
ENSSSCG00000026155	FSCN2	1,58	5,191E-06
ENSSSCG00000023215	MAOB	1,58	5,562E-10
ENSSSCG00000026520	NA	1,58	1,265E-05
ENSSSCG00000010170	DISC1	1,58	1,487E-10
ENSSSCG00000035223	SYNM	1,57	1,001E-07

ENSSSCG00000033189	FAM107A	1,57	5,184E-08
ENSSSCG00000004573	NA	1,57	1,550E-04
ENSSSCG00000031648	CCN5	1,57	1,400E-14
ENSSSCG00000029666	HOXA13	1,56	2,737E-07
ENSSSCG00000006780	WNT2B	1,56	1,971E-12
ENSSSCG00000009216	SPP1	1,56	2,599E-05
ENSSSCG00000004464	TENT5A	1,56	7,696E-05
ENSSSCG00000004822	ALDH1A3	1,55	5,319E-06
ENSSSCG00000004789	THBS1	1,55	5,783E-06
ENSSSCG00000006902	GFI1	1,55	1,150E-03
ENSSSCG00000012257	MAOA	1,55	4,978E-22
ENSSSCG00000010593	CNNM2	1,55	5,421E-03
ENSSSCG00000021973	GPX8	1,54	6,953E-17
ENSSSCG00000010493	PDLIM1	1,54	2,747E-19
ENSSSCG00000025783	ENTPD3	1,54	7,123E-16
ENSSSCG00000002257	MCTP2	1,53	3,586E-11
ENSSSCG00000036941	AKR1E2	1,53	4,684E-17
ENSSSCG00000034351	B3GALT1	1,53	2,211E-13
ENSSSCG00000028031	HDAC11	1,53	5,967E-05
ENSSSCG00000035297	ISG12(A)	1,53	1,054E-08
ENSSSCG00000000519	GLIPR1	1,52	6,925E-05
ENSSSCG00000014184	PAM	1,52	6,067E-10
ENSSSCG00000003705	CABLES1	1,52	7,908E-18
ENSSSCG00000001099	CMAH	1,51	8,465E-09
ENSSSCG00000009468	KCTD12	1,51	3,551E-10
ENSSSCG00000040334	CBX6	1,51	1,001E-15
ENSSSCG00000016684	SCRN1	1,50	7,970E-08
ENSSSCG00000009048	GAB1	1,50	2,805E-16
ENSSSCG00000010143	MTR	1,50	1,956E-14
ENSSSCG00000031199	NA	1,50	7,900E-06
ENSSSCG00000034570	IFI6	1,50	3,998E-06
ENSSSCG00000001421	ZBTB12	1,49	3,691E-05
ENSSSCG00000006169	ZFHX4	1,49	4,895E-16
ENSSSCG00000006038	ZFPM2	1,48	7,493E-09
ENSSSCG00000003459	CELA2A	1,48	3,593E-02
ENSSSCG00000011070	MPP7	1,48	7,489E-07
ENSSSCG00000006928	LMO4	1,48	1,202E-16
ENSSSCG00000028080	ELOVL4	1,47	4,964E-04
ENSSSCG00000035430	YPEL3	1,47	7,578E-14
ENSSSCG00000036553	SAMD12	1,46	6,036E-12
ENSSSCG00000030548	HERC5	1,46	2,617E-10
ENSSSCG00000017886	FBXO39	1,46	4,575E-05

ENSSSCG00000009400	RCBTB2	1,46	4,177E-18
ENSSSCG00000029458	SLC16A2	1,46	2,287E-11
ENSSSCG00000004791	RASGRP1	1,46	5,350E-11
ENSSSCG00000016703	HOXA5	1,46	3,636E-19
ENSSSCG00000004150	NA	1,45	4,434E-04
ENSSSCG00000008073	OMD	1,45	6,745E-05
ENSSSCG00000011643	AMOTL2	1,45	1,461E-10
ENSSSCG00000017754	NA	1,45	1,706E-03
ENSSSCG00000000906	NA	1,44	7,935E-05
ENSSSCG00000013507	MPND	1,44	1,780E-14
ENSSSCG00000035058	PID1	1,44	6,716E-05
ENSSSCG00000015782	IRF2	1,43	2,649E-13
ENSSSCG00000025207	NA	1,43	2,429E-02
ENSSSCG00000038451	NA	1,43	3,489E-02
ENSSSCG00000001910	ISLR	1,43	7,534E-10
ENSSSCG00000027975	LRRC3	1,43	5,239E-03
ENSSSCG00000023362	RHBDF2	1,42	2,458E-10
ENSSSCG00000005250	APBA1	1,42	1,982E-08
ENSSSCG00000037144	CDKN1B	1,42	5,454E-13
ENSSSCG00000013181	SERPING1	1,42	3,094E-11
ENSSSCG00000017904	ENO3	1,42	2,668E-06
ENSSSCG00000025106	NA	1,41	1,733E-05
ENSSSCG00000013020	MAP4K2	1,41	2,881E-05
ENSSSCG00000036418	SLC8A1	1,41	1,845E-04
ENSSSCG00000030408	DDX58	1,41	1,748E-05
ENSSSCG00000009192	PDLIM5	1,40	2,274E-10
ENSSSCG00000032444	PLXDC2	1,40	1,334E-13
ENSSSCG00000036135	COL1A1	1,40	6,039E-06
ENSSSCG00000031707	CTDSP2	1,39	3,924E-13
ENSSSCG00000033479	NEUROG2	1,39	4,441E-03
ENSSSCG00000035012	ELOVL7	1,39	6,109E-06
ENSSSCG00000040779	NA	1,38	6,305E-15
ENSSSCG00000024938	SH3BP5	1,38	2,987E-13
ENSSSCG00000040847	AP1S3	1,38	1,940E-09
ENSSSCG00000039193	HOXB9	1,38	2,755E-09
ENSSSCG00000034997	NA	1,37	5,629E-03
ENSSSCG00000029305	FNDC4	1,37	8,374E-04
ENSSSCG00000014899	PRCP	1,37	4,380E-09
ENSSSCG00000025294	AIRE	1,37	2,026E-04
ENSSSCG00000007472	BCAS4	1,36	8,111E-08
ENSSSCG00000003113	C5AR2	1,36	2,615E-11
ENSSSCG00000003768	NEXN	1,36	4,986E-10

ENSSSCG00000035634	PLSCR1	1,36	1,260E-06
ENSSSCG00000031527	NA	1,36	1,763E-04
ENSSSCG00000040159	NA	1,35	1,071E-03
ENSSSCG00000037262	PTX4	1,35	1,356E-08
ENSSSCG00000032157	GVQW3	1,35	1,623E-05
ENSSSCG00000029249	NAV3	1,35	9,960E-11
ENSSSCG00000011075	KIAA1217	1,35	8,148E-11
ENSSSCG00000033883	NA	1,35	2,243E-05
ENSSSCG00000031723	RAB39A	1,35	3,426E-04
ENSSSCG00000009018	SH3D19	1,34	2,256E-10
ENSSSCG00000024621	KAT2B	1,34	2,611E-15
ENSSSCG00000005626	FPGS	1,34	2,783E-08
ENSSSCG00000000493	FRS2	1,33	1,178E-12
ENSSSCG00000000456	SLC16A7	1,33	3,130E-12
ENSSSCG00000026422	GPER1	1,32	3,294E-03
ENSSSCG00000000649	CLEC1A	1,32	4,423E-10
ENSSSCG00000034949	NA	1,32	2,823E-12
ENSSSCG00000026924	KIFC2	1,32	4,896E-05
ENSSSCG00000009106	PDE5A	1,32	3,439E-13
ENSSSCG00000011147	NA	1,32	1,455E-12
ENSSSCG00000037642	ARID3A	1,31	5,271E-04
ENSSSCG00000022129	ARSE	1,31	5,947E-06
ENSSSCG00000027489	TMCO4	1,31	1,561E-06
ENSSSCG00000027161	PIP5K1B	1,30	8,651E-07
ENSSSCG00000016916	IL6ST	1,30	2,377E-06
ENSSSCG00000036402	MYL9	1,30	6,315E-08
ENSSSCG00000011813	P3H2	1,30	2,633E-08
ENSSSCG00000001755	HYKK	1,30	1,435E-03
ENSSSCG00000039847	C1S	1,30	2,749E-04
ENSSSCG00000016174	FN1	1,29	2,847E-03
ENSSSCG00000009434	RGCC	1,29	1,220E-08
ENSSSCG00000006149	NA	1,29	2,786E-02
ENSSSCG00000008259	LRRTM4	1,29	4,724E-06
ENSSSCG00000030300	MT2A	1,29	7,439E-07
ENSSSCG00000011519	GXYLT2	1,28	2,897E-04
ENSSSCG00000023419	ARHGEF10	1,28	3,965E-15
ENSSSCG00000005308	RUSC2	1,28	1,328E-09
ENSSSCG00000015866	FMNL2	1,27	9,864E-07
ENSSSCG00000008961	MTHFD2L	1,27	4,516E-04
ENSSSCG00000017778	DHRS13	1,27	2,476E-03
ENSSSCG00000040177	ASIC2	1,27	1,425E-02
ENSSSCG00000010494	SORBS1	1,26	7,050E-11

ENSSSCG00000024881	TCP11L2	1,26	1,332E-08
ENSSSCG00000012741	MAMLD1	1,25	5,933E-14
ENSSSCG00000004027	PDE10A	1,25	6,738E-04
ENSSSCG00000001657	CUL7	1,25	3,818E-11
ENSSSCG00000007478	ATP9A	1,25	1,089E-08
ENSSSCG00000009357	SMAD9	1,25	4,813E-07
ENSSSCG00000006069	RGS22	1,25	1,022E-03
ENSSSCG00000008966	PARM1	1,25	5,259E-03
ENSSSCG00000040416	NA	1,25	3,364E-06
ENSSSCG00000022689	GADD45B	1,25	2,676E-10
ENSSSCG00000014993	NA	1,24	3,872E-04
ENSSSCG00000005316	TPM2	1,24	2,007E-07
ENSSSCG00000032367	CEBPD	1,24	3,306E-05
ENSSSCG00000011299	CLEC3B	1,24	2,725E-03
ENSSSCG00000018052	NT5M	1,24	2,370E-02
ENSSSCG00000003708	ANKRD29	1,24	3,373E-08
ENSSSCG00000001860	NRG4	1,24	2,877E-02
ENSSSCG00000012960	CST6	1,24	3,344E-02
ENSSSCG00000011641	SLCO2A1	1,23	3,310E-13
ENSSSCG00000015462	TPK1	1,23	1,650E-10
ENSSSCG00000016706	HOXA2	1,23	1,855E-03
ENSSSCG00000023585	SERINC2	1,23	1,638E-03
ENSSSCG00000039922	CDC42EP5	1,23	2,327E-09
ENSSSCG00000032326	NA	1,22	1,921E-03
ENSSSCG00000034012	CASP3	1,22	5,455E-13
ENSSSCG00000014274	PDLIM4	1,22	1,729E-04
ENSSSCG00000010651	ABLIM1	1,22	3,885E-13
ENSSSCG00000008689	ZFYVE28	1,22	3,168E-03
ENSSSCG00000017920	NA	1,21	1,328E-08
ENSSSCG00000024108	SLC43A2	1,21	3,382E-13
ENSSSCG00000012985	LTBP3	1,21	1,302E-10
ENSSSCG00000005650	CERCAM	1,21	2,824E-10
ENSSSCG00000034491	PRICKLE1	1,21	4,258E-10
ENSSSCG00000013248	LRP4	1,21	3,038E-11
ENSSSCG00000036830	NA	1,21	2,856E-02
ENSSSCG00000005723	NTNG2	1,21	2,195E-03
ENSSSCG00000001513	SYNGAP1	1,21	3,932E-04
ENSSSCG00000017614	TRIM25	1,21	1,691E-07
ENSSSCG00000022202	TOX2	1,20	2,148E-08
ENSSSCG00000006737	IGSF3	1,20	4,631E-05
ENSSSCG00000038842	PCDH9	1,20	1,731E-10
ENSSSCG00000024618	NA	1,20	1,313E-12

ENSSSCG00000016698	HOXA11	1,20	1,309E-07
ENSSSCG00000008820	TEC	1,20	4,603E-10
ENSSSCG00000032200	NA	1,20	4,547E-03
ENSSSCG00000016140	FZD5	1,20	1,058E-05
ENSSSCG00000029571	AVIL	1,19	1,756E-05
ENSSSCG00000013506	STAP2	1,19	5,834E-03
ENSSSCG00000012284	ZNF182	1,19	1,768E-06
ENSSSCG00000008648	RSAD2	1,19	9,834E-03
ENSSSCG00000012916	NA	1,19	1,692E-09
ENSSSCG00000017874	ATP2A3	1,19	1,451E-06
ENSSSCG00000010861	COQ8A	1,19	2,014E-07
ENSSSCG00000037413	KBTBD7	1,19	4,603E-04
ENSSSCG00000008576	NA	1,19	1,048E-06
ENSSSCG00000017569	CHAD	1,19	6,401E-04
ENSSSCG00000006923	GBP2	1,18	1,328E-03
ENSSSCG00000002332	SIPA1L1	1,18	7,495E-10
ENSSSCG00000008020	IFT140	1,17	3,560E-06
ENSSSCG00000003063	PHLDB3	1,17	1,260E-06
ENSSSCG00000003137	PLEKHA4	1,17	1,970E-09
ENSSSCG00000008965	NA	1,17	1,130E-10
ENSSSCG00000012971	EFEMP2	1,17	6,497E-10
ENSSSCG00000011516	EIF4E3	1,17	2,581E-07
ENSSSCG00000010894	TP53BP2	1,16	1,062E-09
ENSSSCG00000039224	CSPG5	1,16	3,070E-05
ENSSSCG00000003828	FGGY	1,16	1,661E-04
ENSSSCG00000035213	ZBTB18	1,16	1,454E-10
ENSSSCG00000030048	PLEKHG2	1,16	8,612E-11
ENSSSCG00000036618	NA	1,16	3,832E-06
ENSSSCG00000009794	MLXIP	1,16	2,155E-12
ENSSSCG00000010456	PANK1	1,16	4,169E-06
ENSSSCG00000013388	PDE3B	1,15	2,259E-10
ENSSSCG00000031875	ZNF469	1,15	8,731E-10
ENSSSCG00000032684	BOK	1,15	5,641E-11
ENSSSCG00000036076	SAPCD1	1,15	4,238E-02
ENSSSCG00000028952	SLC10A6	1,15	9,471E-03
ENSSSCG00000004484	COL12A1	1,15	3,690E-05
ENSSSCG00000014328	GFRA3	1,14	5,646E-10
ENSSSCG00000009881	OAS2	1,14	6,737E-04
ENSSSCG00000036206	DIPK2A	1,14	1,379E-08
ENSSSCG00000012265	CHST7	1,14	4,047E-04
ENSSSCG00000027935	FHOD3	1,14	7,833E-05
ENSSSCG00000002357	ALDH6A1	1,14	5,820E-08

ENSSSCG00000022797	PPP1R3B	1,14	1,928E-05
ENSSSCG00000029533	SEMA4G	1,14	1,385E-04
ENSSSCG00000037312	TMEM234	1,13	2,051E-02
ENSSSCG00000002935	NA	1,13	7,494E-04
ENSSSCG00000021731	WWC2	1,13	9,124E-09
ENSSSCG00000023478	SLC46A1	1,13	5,314E-03
ENSSSCG00000010053	NA	1,13	5,198E-11
ENSSSCG00000035147	NAGLU	1,13	1,672E-10
ENSSSCG00000037905	ITGB5	1,13	6,722E-07
ENSSSCG00000035969	THRA	1,13	2,712E-08
ENSSSCG00000030616	RESF1	1,13	1,087E-10
ENSSSCG00000036190	GIPC3	1,13	5,983E-07
ENSSSCG00000014436	ARHGEF37	1,13	5,550E-10
ENSSSCG00000039186	RAB25	1,13	8,255E-04
ENSSSCG00000017752	WSB1	1,13	1,243E-10
ENSSSCG00000008535	CLIP4	1,12	3,910E-03
ENSSSCG00000015290	CDK18	1,12	9,601E-05
ENSSSCG00000026816	NA	1,12	4,780E-03
ENSSSCG00000037399	NA	1,12	2,274E-03
ENSSSCG00000009475	SLAIN1	1,12	8,616E-05
ENSSSCG00000002932	ZNF567	1,11	6,670E-04
ENSSSCG00000008792	N4BP2	1,11	1,205E-04
ENSSSCG00000016131	ADAM23	1,11	4,620E-03
ENSSSCG00000013294	LDLRAD3	1,11	2,143E-05
ENSSSCG00000015035	C11orf52	1,11	7,592E-03
ENSSSCG00000032687	CYP4V2	1,10	3,344E-06
ENSSSCG00000011699	HPS3	1,10	5,406E-10
ENSSSCG00000006940	CCN1	1,10	1,246E-03
ENSSSCG00000036091	MORC4	1,10	5,341E-07
ENSSSCG00000023791	TMEM229B	1,10	3,072E-02
ENSSSCG00000003081	CEACAM16	1,10	2,118E-03
ENSSSCG00000006197	SULF1	1,10	1,465E-02
ENSSSCG00000008841	PDGFRA	1,10	4,834E-03
ENSSSCG00000006037	OXR1	1,10	1,368E-10
ENSSSCG00000024793	PORCN	1,10	7,071E-03
ENSSSCG00000033919	DCLK1	1,09	1,202E-09
ENSSSCG00000016611	CADPS2	1,09	6,773E-06
ENSSSCG00000024960	PDGFC	1,09	8,264E-08
ENSSSCG00000026618	CAVIN2	1,09	1,223E-08
ENSSSCG00000002664	GSE1	1,09	1,135E-07
ENSSSCG00000014575	NA	1,09	1,113E-02
ENSSSCG00000027628	IL6R	1,09	1,685E-06

ENSSSCG00000005166	MLLT3	1,09	1,126E-06
ENSSSCG00000000156	FBXO7	1,09	8,201E-09
ENSSSCG00000013335	LGR4	1,09	6,084E-03
ENSSSCG00000010483	PLCE1	1,09	7,310E-04
ENSSSCG00000003940	HYI	1,08	6,935E-05
ENSSSCG00000039523	NA	1,08	7,371E-10
ENSSSCG00000011704	WWTR1	1,08	3,340E-11
ENSSSCG00000015036	DIXDC1	1,08	7,892E-04
ENSSSCG00000028190	HOMEZ	1,08	1,091E-07
ENSSSCG00000017219	HID1	1,07	5,479E-09
ENSSSCG00000015322	TFPI2	1,07	1,955E-10
ENSSSCG00000027093	FOLH1B	1,07	1,051E-07
ENSSSCG00000038077	PPP1R14A	1,07	1,013E-08
ENSSSCG00000001620	MDFI	1,07	1,123E-02
ENSSSCG00000022161	ZNF251	1,07	8,030E-08
ENSSSCG00000001780	FAH	1,06	4,949E-06
ENSSSCG00000037647	TFDP2	1,06	2,101E-05
ENSSSCG00000004729	NA	1,06	1,253E-03
ENSSSCG00000002142	TEP1	1,06	3,349E-10
ENSSSCG00000009285	NA	1,06	3,504E-02
ENSSSCG00000000675	C1R	1,06	2,466E-03
ENSSSCG00000011521	PDZRN3	1,05	3,133E-06
ENSSSCG00000033313	C7orf31	1,05	4,710E-02
ENSSSCG00000004379	SOBP	1,05	4,516E-06
ENSSSCG00000021292	OSBPL9	1,04	4,308E-08
ENSSSCG00000033624	SRGAP3	1,04	1,200E-02
ENSSSCG00000010532	LOXL4	1,04	2,178E-07
ENSSSCG00000029805	RHOBTB3	1,04	5,695E-06
ENSSSCG00000006001	ENPP2	1,04	1,330E-04
ENSSSCG00000008647	CMPK2	1,04	5,007E-04
ENSSSCG00000014903	CCDC90B	1,04	1,356E-08
ENSSSCG00000031141	ABCA13	1,04	2,478E-05
ENSSSCG00000035424	NA	1,04	1,027E-02
ENSSSCG00000040162	NUPR1	1,04	4,192E-10
ENSSSCG00000004244	MAN1A1	1,04	1,211E-08
ENSSSCG00000010181	C1orf198	1,04	6,286E-08
ENSSSCG00000012699	NA	1,03	4,379E-08
ENSSSCG00000035791	SIX5	1,03	5,762E-05
ENSSSCG00000011689	PLOD2	1,03	4,826E-06
ENSSSCG00000030560	IGF1R	1,03	3,024E-09
ENSSSCG00000025532	CACNG7	1,03	9,207E-07
ENSSSCG00000003763	IFI44	1,02	1,406E-04

ENSSSCG00000022804	RHOJ	1,02	2,670E-09
ENSSSCG00000040719	KIAA0040	1,02	1,035E-04
ENSSSCG00000004653	SECISBP2L	1,02	1,191E-07
ENSSSCG00000017087	GM2A	1,02	2,194E-05
ENSSSCG00000025049	CYBRD1	1,02	4,507E-10
ENSSSCG00000028108	ASAP3	1,02	5,121E-08
ENSSSCG00000028995	TLE2	1,01	9,456E-08
ENSSSCG00000015001	SLC35F2	1,01	1,135E-09
ENSSSCG00000023010	TMOD1	1,01	1,992E-04
ENSSSCG00000001720	SLC25A27	1,01	1,748E-06
ENSSSCG00000017200	UNC13D	1,01	3,475E-05
ENSSSCG00000004811	PGPEP1L	1,00	2,134E-02
ENSSSCG00000000006	PPARA	1,00	2,099E-03
ENSSSCG00000000910	CRADD	1,00	2,319E-05
ENSSSCG00000001840	KIF7	1,00	1,094E-06
ENSSSCG00000006497	MEX3A	1,00	8,897E-03

Supplemental table 2: Differentially expressed genes in ECs exposed to 80 dyne/cm^2 *vs.* ECs exposed to 30 dyne/cm^2

**Gene_id**	**Gene_name**	**Log$_2$ fold change**	**P-value**
ENSSSCG00000005393	PLPPR1	-4,45	7,259E-20
ENSSSCG00000032878	SHISA9	-4,24	1,782E-23
ENSSSCG00000038783	IGFBP3	-4,12	3,905E-118
ENSSSCG00000015413	FGL2	-2,92	3,654E-27
ENSSSCG00000036201	NPR3	-2,51	7,570E-34
ENSSSCG00000006719	NA	-2,48	1,754E-07
ENSSSCG00000016589	LRRC4	-2,48	6,363E-14
ENSSSCG00000016686	PRR15	-2,31	1,063E-10
ENSSSCG00000015250	ADAMTS15	-2,28	1,096E-12
ENSSSCG00000034943	GDF6	-2,27	7,763E-42
ENSSSCG00000004464	TENT5A	-2,26	7,267E-11
ENSSSCG00000014310	CXCL14	-2,13	5,378E-06
ENSSSCG00000006051	CTHRC1	-2,07	1,012E-09
ENSSSCG00000016174	FN1	-2,02	1,240E-15
ENSSSCG00000009222	SPARCL1	-2,01	5,083E-11
ENSSSCG00000002997	NA	-2,00	1,754E-04
ENSSSCG00000032151	CPZ	-1,90	1,287E-38
ENSSSCG00000016034	COL3A1	-1,90	8,557E-15
ENSSSCG00000006197	SULF1	-1,81	9,366E-11
ENSSSCG00000038866	NA	-1,77	5,112E-03
ENSSSCG00000036418	SLC8A1	-1,77	4,154E-18
ENSSSCG00000031524	NA	-1,74	9,757E-08
ENSSSCG00000028549	ECM2	-1,74	9,139E-13
ENSSSCG00000003439	DHRS3	-1,73	3,007E-14
ENSSSCG00000035858	NA	-1,69	4,388E-02
ENSSSCG00000012818	F8	-1,69	5,904E-37
ENSSSCG00000022129	ARSE	-1,64	7,601E-07
ENSSSCG00000039464	NA	-1,64	3,026E-03
ENSSSCG00000011129	ITIH5	-1,63	3,354E-16
ENSSSCG00000000456	SLC16A7	-1,61	1,178E-19
ENSSSCG00000036049	PEG10	-1,60	3,557E-24
ENSSSCG00000011756	NLGN1	-1,58	2,299E-08
ENSSSCG00000008749	SLIT2	-1,58	3,275E-09
ENSSSCG00000036033	THRB	-1,54	3,872E-07
ENSSSCG00000035720	HRCT1	-1,54	7,422E-09
ENSSSCG00000014975	NA	-1,52	1,963E-08
ENSSSCG00000031960	NA	-1,52	1,982E-09
ENSSSCG00000011110	CCDC3	-1,52	2,024E-03
ENSSSCG00000015326	COL1A2	-1,49	4,161E-06

ENSSSCG00000000010	FBLN1	-1,49	3,251E-34
ENSSSCG00000011641	SLCO2A1	-1,48	6,358E-23
ENSSSCG00000015610	SYT14	-1,47	5,374E-04
ENSSSCG00000034993	NREP	-1,40	5,393E-07
ENSSSCG00000005203	IL33	-1,39	1,616E-22
ENSSSCG00000032423	SPOCK1	-1,38	4,690E-03
ENSSSCG00000005627	AK1	-1,37	3,246E-11
ENSSSCG00000011463	IL17RD	-1,37	1,636E-04
ENSSSCG00000009114	PRSS12	-1,35	1,914E-26
ENSSSCG00000002314	SMOC1	-1,34	4,125E-07
ENSSSCG00000001910	ISLR	-1,32	5,200E-26
ENSSSCG00000011609	FBLN2	-1,31	1,380E-09
ENSSSCG00000007073	ISM1	-1,29	1,954E-05
ENSSSCG00000021562	PLXNA4	-1,29	1,217E-08
ENSSSCG00000037330	NA	-1,29	4,049E-02
ENSSSCG00000032251	MAFB	-1,26	1,082E-03
ENSSSCG00000015175	VWA5A	-1,26	3,691E-20
ENSSSCG00000036135	COL1A1	-1,25	2,342E-03
ENSSSCG00000017296	ACE	-1,25	1,309E-13
ENSSSCG00000031503	PRRX1	-1,24	8,537E-10
ENSSSCG00000023305	NA	-1,24	9,558E-04
ENSSSCG00000016866	GHR	-1,24	1,808E-09
ENSSSCG00000037241	RGS2	-1,23	1,525E-06
ENSSSCG00000028931	GDF7	-1,23	1,214E-12
ENSSSCG00000030515	GABRA4	-1,23	1,157E-03
ENSSSCG00000032241	GPNMB	-1,23	1,241E-16
ENSSSCG00000031119	NA	-1,22	2,120E-06
ENSSSCG00000006418	NA	-1,21	1,177E-08
ENSSSCG00000038956	HEYL	-1,21	1,171E-03
ENSSSCG00000033624	SRGAP3	-1,20	6,171E-06
ENSSSCG00000013333	BDNF	-1,20	9,392E-04
ENSSSCG00000000647	OLR1	-1,19	1,549E-17
ENSSSCG00000011522	CNTN3	-1,19	7,648E-16
ENSSSCG00000014328	GFRA3	-1,18	1,805E-12
ENSSSCG00000011973	COL8A1	-1,17	2,508E-10
ENSSSCG00000031831	CADM1	-1,16	1,964E-17
ENSSSCG00000005277	GCNT1	-1,15	1,752E-15
ENSSSCG00000039847	C1S	-1,15	1,196E-05
ENSSSCG00000001457	SLA-DQB1	-1,15	2,719E-02
ENSSSCG00000016225	MOGAT1	-1,14	5,683E-04
ENSSSCG00000038842	PCDH9	-1,14	4,364E-10
ENSSSCG00000008648	RSAD2	-1,14	7,306E-07

ENSSSCG00000006321	FAM78B	-1,13	2,873E-08
ENSSSCG00000030921	APOA1	-1,12	2,278E-16
ENSSSCG00000024206	CPPED1	-1,10	1,343E-11
ENSSSCG00000002275	PPP1R36	-1,09	1,171E-03
ENSSSCG00000034570	IFI6	-1,09	6,204E-10
ENSSSCG00000006161	IL7	-1,09	1,688E-07
ENSSSCG00000006624	SELENBP1	-1,08	1,865E-03
ENSSSCG00000004570	TPM1	-1,08	1,290E-08
ENSSSCG00000032709	ARL4A	-1,08	1,376E-12
ENSSSCG00000023215	MAOB	-1,08	1,537E-04
ENSSSCG00000035595	HMCN1	-1,07	9,600E-13
ENSSSCG00000000649	CLEC1A	-1,07	7,240E-07
ENSSSCG00000005455	SVEP1	-1,07	2,407E-12
ENSSSCG00000011495	PRICKLE2	-1,07	4,928E-10
ENSSSCG00000032181	C17orf58	-1,07	6,809E-04
ENSSSCG00000011519	GXYLT2	-1,07	6,815E-04
ENSSSCG00000035297	ISG12(A)	-1,07	2,125E-09
ENSSSCG00000030843	APLN	-1,06	2,444E-04
ENSSSCG00000000778	CPNE8	-1,05	5,720E-04
ENSSSCG00000016685	WIPF3	-1,05	6,302E-03
ENSSSCG00000030300	MT2A	-1,05	1,982E-09
ENSSSCG00000037697	MGP	-1,05	1,321E-13
ENSSSCG00000011077	NA	-1,04	7,925E-04
ENSSSCG00000039573	SLPI	-1,04	1,052E-10
ENSSSCG00000012448	ITM2A	-1,03	2,263E-10
ENSSSCG00000034887	HAPLN4	-1,03	1,004E-16
ENSSSCG00000012510	ARMCX2	-1,02	1,201E-03
ENSSSCG00000001716	RCAN2	-1,02	6,360E-03
ENSSSCG00000011043	C1QL3	-1,01	3,351E-03
ENSSSCG00000025836	SULT1C4	-1,01	1,643E-09
ENSSSCG00000003826	HOOK1	-1,01	6,789E-04
ENSSSCG00000033577	MED30	-1,00	6,204E-10
ENSSSCG00000011195	GALNT15	-1,00	5,415E-03
ENSSSCG00000032094	DKK2	5,31	2,157E-19
ENSSSCG00000023165	SEMA7A	3,19	1,300E-27
ENSSSCG00000003539	GRHL3	3,16	6,338E-13
ENSSSCG00000012026	ADAMTS1	2,81	1,762E-32
ENSSSCG00000003616	FAM167B	2,67	1,284E-12
ENSSSCG00000008427	KCNK12	2,58	7,217E-08
ENSSSCG00000010325	KCNMA1	2,55	7,989E-47
ENSSSCG00000015930	DHRS9	2,49	5,108E-18
ENSSSCG00000006472	CRABP2	2,46	1,569E-36

ENSSSCG00000026852	NPPC	2,37	1,816E-29
ENSSSCG00000004980	THSD4	2,37	6,493E-30
ENSSSCG00000002825	NA	2,34	7,077E-17
ENSSSCG00000038080	EMCN	2,34	1,030E-20
ENSSSCG00000024158	ANO1	2,31	3,251E-34
ENSSSCG00000024312	ID4	2,29	4,538E-05
ENSSSCG00000017700	CCL3L1	2,27	5,372E-05
ENSSSCG00000003600	TINAGL1	2,26	3,251E-34
ENSSSCG00000008118	PROM2	2,22	1,009E-10
ENSSSCG00000010199	RET	2,21	2,258E-19
ENSSSCG00000035524	WNT9B	2,07	6,547E-47
ENSSSCG00000038929	CEMIP	2,07	6,722E-51
ENSSSCG00000015556	LAMC2	2,05	1,914E-26
ENSSSCG00000032838	MYOZ2	2,04	1,709E-07
ENSSSCG00000033190	NA	2,03	1,914E-26
ENSSSCG00000036801	C6orf132	1,99	3,977E-16
ENSSSCG00000009477	EDNRB	1,96	1,793E-07
ENSSSCG00000010449	CH25H	1,83	6,835E-23
ENSSSCG00000031321	NR4A1	1,82	7,808E-28
ENSSSCG00000022256	DEPP1	1,78	2,969E-14
ENSSSCG00000010607	COL17A1	1,75	4,983E-16
ENSSSCG00000023591	ADGRF2	1,71	8,179E-20
ENSSSCG00000016295	NGEF	1,71	6,352E-14
ENSSSCG00000015550	RGS16	1,69	8,743E-23
ENSSSCG00000006595	IVL	1,64	4,687E-05
ENSSSCG00000016068	HECW2	1,61	5,968E-13
ENSSSCG00000010479	RBP4	1,60	1,493E-05
ENSSSCG00000035417	TMEM238	1,59	8,477E-03
ENSSSCG00000037416	CLIC5	1,53	1,998E-06
ENSSSCG00000023737	CSF2	1,53	6,721E-15
ENSSSCG00000006735	PTGFRN	1,51	3,212E-17
ENSSSCG00000009000	NA	1,51	8,996E-06
ENSSSCG00000034858	RAP1GAP2	1,51	2,205E-13
ENSSSCG00000008115	NA	1,51	2,873E-05
ENSSSCG00000005707	FIBCD1	1,49	2,663E-10
ENSSSCG00000040295	NA	1,47	2,705E-04
ENSSSCG00000008888	NPY1R	1,47	3,608E-13
ENSSSCG00000027911	LTB4R	1,47	1,237E-04
ENSSSCG00000021515	HS3ST1	1,47	5,024E-10
ENSSSCG00000004191	MOXD1	1,47	1,594E-06
ENSSSCG00000021259	CDA	1,47	5,325E-27
ENSSSCG00000005287	PSAT1	1,46	6,691E-05

ENSSSCG00000008318	VAX2	1,45	1,323E-08
ENSSSCG00000038610	INHBB	1,45	7,079E-03
ENSSSCG00000024837	SYT12	1,43	1,595E-13
ENSSSCG00000003694	EMILIN2	1,42	4,023E-19
ENSSSCG00000035224	NA	1,42	1,165E-13
ENSSSCG00000006359	ADAMTS4	1,39	4,790E-18
ENSSSCG00000021585	OXTR	1,39	1,622E-07
ENSSSCG00000007864	GPRC5B	1,39	4,306E-22
ENSSSCG00000012996	CDC42EP2	1,38	4,118E-09
ENSSSCG00000013880	NA	1,38	3,516E-19
ENSSSCG00000010381	ARHGAP22	1,38	6,171E-06
ENSSSCG00000026984	DIPK2B	1,37	6,204E-10
ENSSSCG00000011596	TRH	1,37	9,635E-10
ENSSSCG00000013976	NA	1,37	1,081E-06
ENSSSCG00000000261	IGFBP6	1,37	7,100E-17
ENSSSCG00000038727	GDNF	1,37	2,043E-02
ENSSSCG00000011837	MELTF	1,35	4,549E-04
ENSSSCG00000035256	NA	1,34	6,746E-08
ENSSSCG00000005036	GPR137C	1,33	1,598E-05
ENSSSCG00000022961	CLMP	1,33	3,110E-07
ENSSSCG00000023522	TGM2	1,32	9,855E-25
ENSSSCG00000003797	DIRAS3	1,31	1,843E-04
ENSSSCG00000039986	RGS8	1,31	2,770E-05
ENSSSCG00000008319	CD207	1,31	7,538E-05
ENSSSCG00000032374	SULT1B1	1,31	2,541E-16
ENSSSCG00000028076	ZBTB7C	1,30	1,314E-08
ENSSSCG00000013252	F2	1,30	2,935E-10
ENSSSCG00000000188	DHH	1,30	3,255E-10
ENSSSCG00000012027	ADAMTS5	1,28	2,670E-05
ENSSSCG00000033286	NA	1,28	3,298E-11
ENSSSCG00000014213	KCNN2	1,28	2,361E-04
ENSSSCG00000003431	NPPB	1,27	1,154E-03
ENSSSCG00000017993	NTN1	1,26	4,154E-10
ENSSSCG00000038521	CHAC1	1,26	3,257E-12
ENSSSCG00000009182	NA	1,25	1,872E-03
ENSSSCG00000029592	GPRC5A	1,25	8,112E-18
ENSSSCG00000021711	C16orf86	1,24	1,862E-04
ENSSSCG00000009083	SPRY1	1,23	5,455E-10
ENSSSCG00000007039	PRNP	1,23	3,205E-14
ENSSSCG00000003762	ADGRL4	1,22	2,263E-15
ENSSSCG00000015664	NA	1,21	4,357E-21
ENSSSCG00000028661	ENKD1	1,21	3,363E-04

ENSSSCG00000017306	ITGB3	1,21	8,522E-24
ENSSSCG00000035249	GADD45G	1,21	8,259E-09
ENSSSCG00000027677	NA	1,21	3,088E-13
ENSSSCG00000006664	MTMR11	1,21	1,217E-03
ENSSSCG00000004670	C15orf48	1,21	7,853E-11
ENSSSCG00000009929	TRPV4	1,20	3,382E-08
ENSSSCG00000000699	LPAR5	1,19	5,657E-05
ENSSSCG00000007147	HSPA12B	1,18	1,452E-07
ENSSSCG00000005688	PTGES	1,18	1,392E-11
ENSSSCG00000008618	MYCN	1,17	3,062E-08
ENSSSCG00000033566	NA	1,17	1,167E-03
ENSSSCG00000029331	PALLD	1,17	5,820E-04
ENSSSCG00000010312	PLAU	1,17	1,491E-16
ENSSSCG00000039862	TRIB3	1,17	1,052E-10
ENSSSCG00000012490	TMEM35A	1,16	1,835E-12
ENSSSCG00000010370	NA	1,15	2,663E-14
ENSSSCG00000032434	PLAUR	1,15	2,785E-17
ENSSSCG00000040110	NA	1,14	5,585E-04
ENSSSCG00000001867	PSTPIP1	1,14	1,671E-05
ENSSSCG00000005094	TMEM30B	1,13	4,529E-07
ENSSSCG00000001518	ITPR3	1,13	2,059E-18
ENSSSCG00000008147	FHL2	1,11	2,708E-14
ENSSSCG00000003080	NA	1,11	1,546E-12
ENSSSCG00000003069	KCNN4	1,10	6,965E-14
ENSSSCG00000001917	CD276	1,10	2,446E-15
ENSSSCG00000029199	SCN4B	1,09	5,447E-03
ENSSSCG00000006296	ATP1B1	1,07	1,795E-15
ENSSSCG00000004291	NT5E	1,07	6,194E-04
ENSSSCG00000036437	NOG	1,07	1,079E-10
ENSSSCG00000023716	TNFAIP6	1,07	1,763E-04
ENSSSCG00000016548	NA	1,06	1,961E-07
ENSSSCG00000036824	AVPI1	1,06	3,423E-11
ENSSSCG00000006183	SBSPON	1,05	1,781E-04
ENSSSCG00000040366	ADAMTSL1	1,05	1,016E-05
ENSSSCG00000008240	NA	1,03	5,115E-05
ENSSSCG00000017052	ADAM19	1,03	8,042E-16
ENSSSCG00000006578	S100A4	1,02	2,550E-13
ENSSSCG00000036905	NA	1,02	5,722E-03
ENSSSCG00000034863	PARD6A	1,02	8,781E-05
ENSSSCG00000008038	SLC9A3R2	1,01	6,678E-08
ENSSSCG00000029752	C16orf54	1,01	1,987E-02

Supplemental table 3: Genes with an up- or down- regulated expression in the 8 following protein classes: Cell adhesion molecules, Cell junction proteins, Cytoskeletal proteins, Extracellular matrix proteins, Intercellular signal molecules, Scaffold/adaptor proteins, Structural proteins and Transmembrane signal receptors

**Protein class**	**Gene name**<br>**2 *vs*. 30 dyne/cm^2**	**Gene name**<br>**80 *vs*. 30 dyne/cm^2**	**Gene name**<br>**2 *vs*. 30 dyne/cm^2**<br>**and**<br>**80 *vs*. 30 dyne/cm^2**
Cell adhesion molecules (PC00069)	ITGA5		ITGB3
	TGFBI		PCDH9
	ITGA10		ISLR
	ITGA6		NLGN1
	PCDH12		
	POSTN		
	THY1		
	ITGA1		
	CDH24		
	SPON1		
	ITGB5		
Cell junction proteins (PC00070)	CLDN9		PARD6A
	CLDN22		
	GJC1		
	KIF12		
Cytoskeletal proteins (PC00085)	KIF12	MYOZ2	PSTPIP1
	PDLIM4		
	MYL9		
	EZR		
	ANLN		
	KIFC2		
	ABLIM1		
	FSCN2		
	PDLIM5		
	PDLIM1		
	SH3YL1		
	MICAL1		
	TPM2		
	RTKN2		
	KIF21A		
	CAPG		
	WASF3		
	TNS3		
	AVIL		
	SHROOM4		
	EMP1		
	CORO2A		
	TCP11L2		
	SYNPO2		

	PLEK2		
	SGCG		
	PDLIM7		
	EMP3		
	KIF7		
Extracellular matrix proteins (PC00102)	STOM	FBLN2	COL1A1
	NTNG2	COL1A2	SPOCK1
	COL23A1	HAPLN4	COL3A1
	COL26A1	COL8A1	LAMC2
	LTBP1	COL17A1	SPARCL1
	P3H2		FBLN1
	EFEMP1		
	EFEMP2		
	LTBP3		
	COL12A1		
	COLQ		
	MATN4		
Intercellular signal molecules (PC00207)	CCN1	CSF2	RCAN2
	CCL11	SEMA7A	GPNMB
	SEMA4G	INHBB	BDNF
	NRG1	CCL3L1	WNT9B
	NXPH3	GDNF	GDF7
	EDN1		NOG
	CLEC3B		FN1
	STC2		FIBCD1
	CCN5		GDF6
	INHBA		NPPC
	WNT2B		FGL2
	ANGPTL5		CXCL14
	SPP1		
	BMP4		
	PTHLH		
	IL11		
	EFNB1		
	ANGPTL4		
	ANGPT2		
	ADM		
	CCL2		
	SEMA3G		
	CXCL10		
	EFNA4		
	PDGFC		
	IL34		
	CCN2		
Scaffold/adaptor proteins (PC00226)	CCN3		PRICKLE2
	LMCD1		SPRY1
	KBTBD7		

	GULP1		
	FRY		
	RASSF3		
	STAP2		
	KLHL5		
	TRAF1		
	ANKRD50		
	ANKRD28		
	KLHL1		
	GIPC3		
	KCTD15		
	SH3BP5		
	DIXDC1		
	SH2B3		
	ANK2		
	RASSF2		
	SWAP70		
	CKS2		
	GAB1		
	NPTN		
	KCTD12		
	CASKIN2		
	KCTD7		
	YWHAH		
	DAPK1		
	AKAP12		
	NEXN		
	PRICKLE1		
	MPP7		
Structural proteins (PC00211)	ELN		SCN4B
	COLEC10		
	IFT140		
Transmembrane signal receptors (PC00197)	TNFRSF21	LTB4R	PLAUR
	GPR15	OXTR	NPR3
	FZD5	GPRC5B	ADGRL4
	MERTK		LPAR5
	PDGFRA		IL17RD
	IL6ST		NPY1R
	F2RL3		GPRC5A
	RAMP2		RET
	GPER1		
	GPR150		
	GIPR		
	CD40		
	GPR158		
	FLT1		

	IL20RA		
	ADORA2A		
	F2R		
	FGFRL1		
	LRRN2		
	EPHB1		
	LGR4		
	IL10RA		
	GPR4		
	PTGIR		
	TEK		
	SDC1		
	KDR		
	FZD8		
	SIGIRR		
	CHAD		
	OSMR		
	F2RL1		
	C5AR2		
	RTN4RL1		
	P2RY1		
	P2RY2		
	LRIG1		
	ADRB2		
	LYPD5		
	CALCR		
	NYX		
	TNFRSF11A		
